# STING agonist 8803 reprograms the immune microenvironment and increases survival in preclinical models of glioblastoma

**DOI:** 10.1172/JCI175033

**Published:** 2024-06-17

**Authors:** Hinda Najem, Spencer T. Lea, Shashwat Tripathi, Lisa Hurley, Chao-Hsien Chen, Ivana William, Moloud Sooreshjani, Michelle Bowie, Genevieve Hartley, Corey Dussold, Sebastian Pacheco, Crismita Dmello, Catalina Lee-Chang, Kathleen McCortney, Alicia Steffens, Jordain Walshon, Martina Ott, Jun Wei, Anantha Marisetty, Irina Balyasnikova, Roger Stupp, Rimas V. Lukas, Jian Hu, Charles David James, Craig M. Horbinski, Maciej S. Lesniak, David M. Ashley, Waldemar Priebe, Leonidas C. Platanias, Michael A. Curran, Amy B. Heimberger

**Affiliations:** 1 Department of Neurological Surgery and; 2 Malnati Brain Tumor Institute of the Robert H. Lurie Comprehensive Cancer Center, Feinberg School of Medicine, Northwestern University, Chicago, Illinois, USA.; 3 Department of Immunology, The University of Texas MD Anderson Cancer Center, Houston, Texas, USA.; 4 Department of Neurology, Houston Methodist Neurological Institute, Houston, Texas, USA.; 5 Department of Medicine, Duke University School of Medicine, Durham, North Carolina, USA.; 6 Miltenyi Biotec, Bergisch Gladbach, Germany.; 7 Immatics US, Houston, Texas, USA.; 8 Department of Neurology, Feinberg School of Medicine, Northwestern University, Chicago, Illinois, USA.; 9 Department of Cancer Biology and; 10 Department of Experimental Therapeutics, Division of Cancer Medicine, The University of Texas MD Anderson Cancer Center, Houston, Texas, USA.; 11 Moleculin, Houston, Texas, USA.; 12 Robert H. Lurie Comprehensive Cancer Center and Division of Hematology-Oncology, Department of Medicine, Feinberg School of Medicine, Northwestern University, Chicago, Illinois, USA.

**Keywords:** Immunology, Oncology, Brain cancer, Cancer immunotherapy, Signal transduction

## Abstract

STING agonists can reprogram the tumor microenvironment to induce immunological clearance within the central nervous system. Using multiplexed sequential immunofluorescence (SeqIF) and the Ivy Glioblastoma Atlas, STING expression was found in myeloid populations and in the perivascular space. The STING agonist 8803 increased median survival in multiple preclinical models of glioblastoma, including QPP8, an immune checkpoint blockade–resistant model, where 100% of mice were cured. Ex vivo flow cytometry profiling during the therapeutic window demonstrated increases in myeloid tumor trafficking and activation, alongside enhancement of CD8^+^ T cell and NK effector responses. Treatment with 8803 reprogrammed microglia to express costimulatory CD80/CD86 and iNOS, while decreasing immunosuppressive CD206 and arginase. In humanized mice, where tumor cell STING is epigenetically silenced, 8803 therapeutic activity was maintained, further attesting to myeloid dependency and reprogramming. Although the combination with a STAT3 inhibitor did not further enhance STING agonist activity, the addition of anti–PD-1 antibodies to 8803 treatment enhanced survival in an immune checkpoint blockade–responsive glioma model. In summary, 8803 as a monotherapy demonstrates marked in vivo therapeutic activity, meriting consideration for clinical translation.

## Abstract 2

STING agonist 8803 induces effector responses in T cells and NK cells and mobilizes inflammatory myeloid cells, without inducing CNS autoimmunity in preclinical models of glioblastoma.

## Introduction

The prognosis of glioblastoma patients is poor, with an approximate median overall survival of 21 months (1). Whereas radiation and chemotherapy are standard treatments at the time of diagnosis, there are no effective therapies for recurrence (2). Immunotherapy for cancer has shown tremendous progress in achieving long-term remissions in many other cancers, even in advanced metastatic disease, and is an accepted standard of care for multiple tumor types (3). In contrast, immunotherapy has not shown efficacy in most glioblastoma patients (4) secondary to diverse and redundant mechanisms of intrinsic and iatrogenic immunosuppression, downregulation of antigen processing and presentation, and a paucity of T cells in the tumor (5–10).

Recent studies showed that activation of the stimulator of interferon (IFN) genes (STING) pathway increases antitumoral immune responses in gliomas and melanoma (11). STING senses the presence of double-stranded DNA and triggers the release of IFN from myeloid cells/macrophages, which are major components of the tumor microenvironment (TME) in gliomas (12, 13). In comparison with other innate immune agonists, STING activation can re-educate immunosuppressive macrophages toward a proinflammatory state and can reverse the suppressive properties of myeloid-derived suppressor cells (MDSCs) (14, 15). Furthermore, mouse gliomas grow at a faster rate in STING-knockout mice, demonstrating the key role of this pathway in limiting tumor progression (11). STING agonists are particularly compelling therapeutics for gliomas because they can trigger T cell infiltration into immunologically “cold” tumors through proinflammatory activation of an immunosuppressive TME. STING agonists have been selected for translational clinical studies for the following reasons: (a) they stimulate a foreign body reaction, (b) they induce IFN-γ production essential for T cell effector action, (c) they induce chemokine-induced T cell trafficking to the tumor, and (d) they are scalable, inexpensive, and easy to generate as a clinical product. Prior studies have demonstrated the capacity for intratumorally injected STING agonists to eliminate the treated tumor, including distant untreated sites of disease (16). STING agonists are sufficiently potent to induce complete tumor response in canines with spontaneously arising high-grade gliomas (12).

The signal transducer and activator of transcription 3 (STAT3) pathway regulates gliomagenesis (17) and is a key hub of tumor-mediated immunosuppression (18). STAT3 is a transcription factor that is activated through phosphorylation induced by a variety of signals such as IL-6. Activated STAT3 inhibits the production of proinflammatory cytokines that are necessary for the maturation of dendritic cells (DCs) and the generation of effector T cells (19). STAT3 is activated in various immune cell populations by tumor-elaborated products, resulting in profound immunosuppression. WP1066, a blood–brain-barrier penetrant caffeic acid analog that blocks the nuclear translocation of p-STAT3 (20), has demonstrated minimal toxicity in phase I testing (21) and will be used with radiation in an upcoming phase II clinical trial (ClinicalTrials.gov NCT05879250) based on preclinical modeling (22). WP1066 also has substantial immunomodulatory properties, including inducing the expression of costimulatory molecules on peripheral macrophages, inducing T cell proliferation and effector responses, and inhibiting Tregs. Theoretically, inhibiting an immunosuppressive hub and tumor driver such as STAT3, paired with an activator of the immune system such as a STING agonist, should generate potent immunological responses.

A major limitation of using immune checkpoint inhibitors such as anti–PD-1 antibodies in gliomas is the low frequency of T cell effector responses in the TME, and as such, the target for such agents to act upon. Four key steps are needed to generate an antitumor immune response: (a) an immunological target, (b) immune activation, (c) immune trafficking to the TME, and (d) maintenance of an effector response. Combinations of immunomodulatory agents in vivo can alter the balance of immunostimulation and immunosuppression within the glioma TME to favor immune activation and targeted clearance of tumor. The recent development of a potent, clinical-grade STING agonist (15, 23) offers the opportunity to explore whether STING activation is an effective immunotherapy for gliomas, and whether this treatment can be further enhanced by combining it with immunomodulators such as STAT3 blockade (24) or anti–PD-1 therapy.

## Results

### Spatial location of STING and STAT3 in the glioblastoma TME.

**Figure 1:**
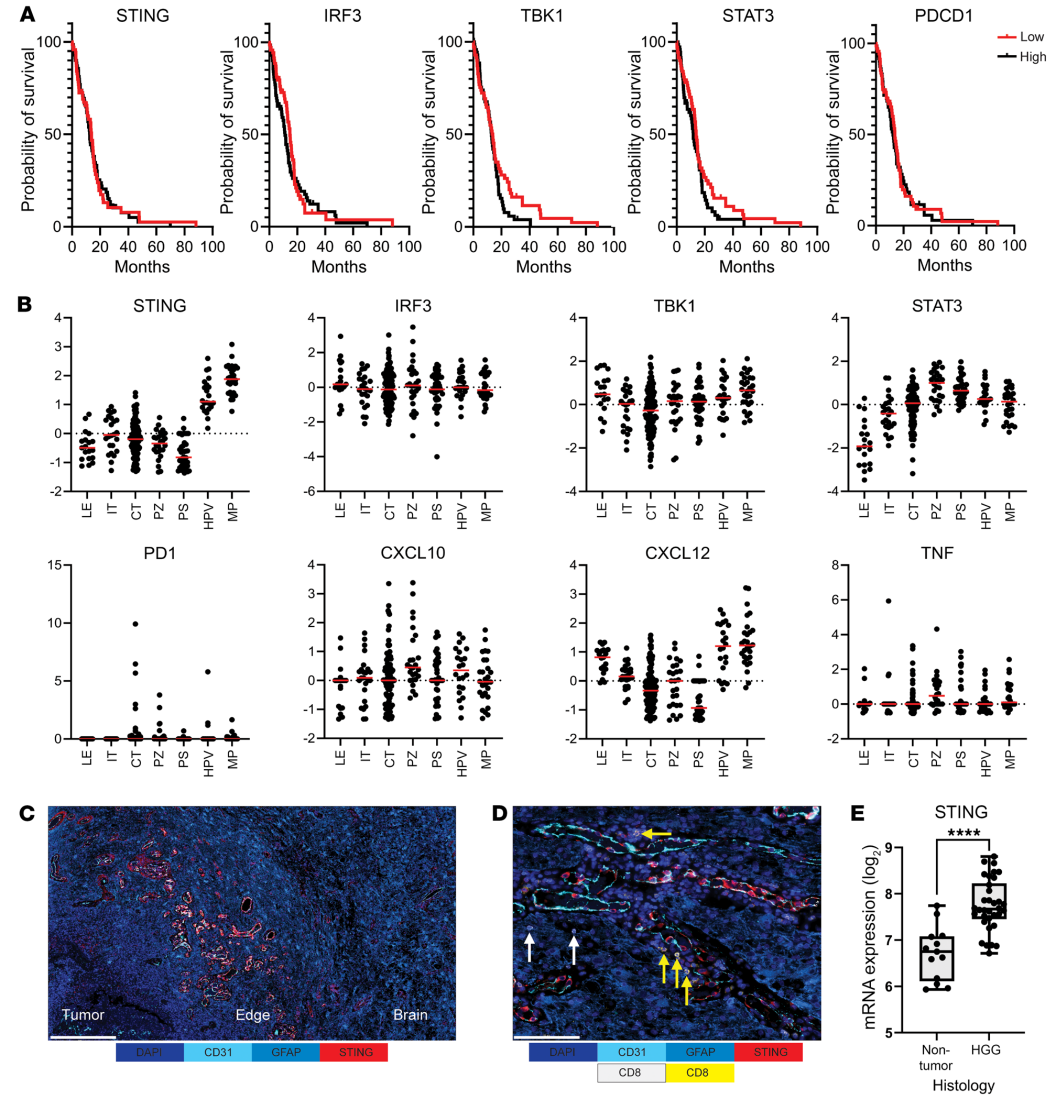
The prognostic impact and localization of the STING pathway in human glioblastoma. (**A**) Kaplan-Meier survival curves of newly diagnosed IDH-1–WT glioblastoma patients stratified based on high versus low expression stratified on the median of the designated marker. STING (*TMEM173*): high (*n* = 71, events = 62; median = 12.6); low (*n* = 71, events = 54, median = 13.8); HR = 1 (0.69–1.45); log-rank *P* value = 0.99; Wilcoxon’s *P* value = 0.92. IRF3: high (*n* = 71; events = 62; median = 11.2); low (*n* = 71, events = 54; median = 14.7); HR = 0.84 (0.58–1.22); log-rank *P* value = 0.37; Wilcoxon’s *P* value = 0.08. Tbk1: high (*n* = 72, events = 57, median = 12.9); low (*n* = 70, events = 59, median = 13.8); HR = 0.74 (0.51–1.08); log-rank *P* value = 0.12; Wilcoxon’s *P* value = 0.67. STAT3: high (*n* = 72, events = 57, median = 11.8); low (*n* = 70, events = 59, median = 13.8); HR = 0.74 (0.51–1.08); log-rank *P* value = 0.11; Wilcoxon’s *P* value = 0.23. PD-1 (PDCD1): high (*n* = 72, events = 57, median = 12.3); low (*n* = 70, events = 59, median = 13.8); HR = 0.94 (0.65–1.36); log-rank *P* value = 0.76; Wilcoxon’s *P* value = 0.72. (**B**) RNA sequencing data from the Ivy Glioblastoma Atlas project was analyzed based on differences in the anatomical locations of these markers in primary gliomas. The *y* axes show *z* score–normalized mRNA expression. LE, leading edge; IT, infiltrating tumor; CT, cellular tumor; PZ, perinecrotic zone; PS, pseudopalisading cells around necrosis; HPV, hyperplastic blood vessels in cellular tumor; MP, microvascular proliferation. (**C**) Representative multiplexed sequential immunofluorescence (SeqIF) imaging of human glioblastoma showing the transition of the microenvironment from tumor to brain, with the highest expression of perivascular STING at the edge. Color panel: DAPI, dark blue; CD31, cyan blue; GFAP, blue; and STING, red. Scale bar: 500 μm. (**D**) Representative multiplexed SeqIF imaging of human glioblastoma, demonstrating the confinement of T cells to the perivascular regions of CD31^+^ vessels, as described and quantified in the spatial bioinformatic analysis protocol by Najem et al. (57). Color panel: DAPI, dark blue; CD31, cyan blue; GFAP, blue; STING, red; CD4, yellow; and CD8, white. Yellow arrows indicate CD4^+^ T cells and white arrows indicate CD8^+^ T cells. Scale bar: 100 μm. (**E**) Analysis of mRNA STING expression in non–tumor-bearing brain relative to high-grade glioma (HGG) (27). Box-and-whisker plots show the minimum and maximum; lines represent 25%, median, and 75%. For non-tumor, min: 5.936, max: 7.743, 25%: 6.113, 75%: 7.083, median: 6.753. For HGG, min: 6.715, max: 8.158, 25%: 7.451, 75%: 8.229, median: 7.668. ****P < 0.0001 (2-tailed Student’s *t* test).

To clarify whether the expression of STING or STAT3 is prognostic for outcomes in glioblastoma, we analyzed the newly diagnosed isocitrate dehydrogenase 1–WT (IDH-1–WT) cohort from The Cancer Genome Atlas (TCGA). There was no significant difference in glioblastoma patient prognosis as a function of *STAT3*, PD-1 (*PDCD1*), STING (*TMEM173*), and downstream *IRF3* (IFN regulatory factor 3) and *TBK1* (tank-binding kinase 1) (25, 26) RNA expression (Figure 1A). Using the Ivy Glioblastoma Atlas as we have previously described (24), the expression of STING was found to be increased in the hyperplastic blood vessels (HPVs) and microvasculature proliferation (MP) regions (Figure 1B). The STING pathway did not appear to be activated in these regions, since the downstream expression of *IRF3* and *TBK1* was not elevated. *STAT3* expression was associated with the perinecrotic, necrotic, and vascular areas. This was distinct from the cellular tumor regions in which only low levels of PD-1 could be detected in the TME (Figure 1B). Using multiplexed sequential immunofluorescence (SeqIF), the expression of STING was validated on the HPVs and MP regions within the glioblastoma (Figure 1C). STING expression in the blood vessels was the highest at the interface between the glioblastoma and the brain (Figure 1C). The T cell chemokine *CXCL12* was also enriched in these vascular regions (Figure 1B) and when rare T cells were present, they were typically located in the perivascular regions, especially at the tumor edge (Figure 1D). These data indicate that there are specific differences in the immunoreactivity of the glioblastoma vasculature relative to adjacent normal brain. Bioinformatic analysis of a pediatric glioma database (27), which contains normal brain as a comparator, demonstrates that STING mRNA expression is lower in non–tumor-bearing brains relative to the levels in high-grade glioma (Figure 1E).

### STING agonists demonstrate therapeutic activity in preclinical models of glioblastoma.

**Figure 2:**
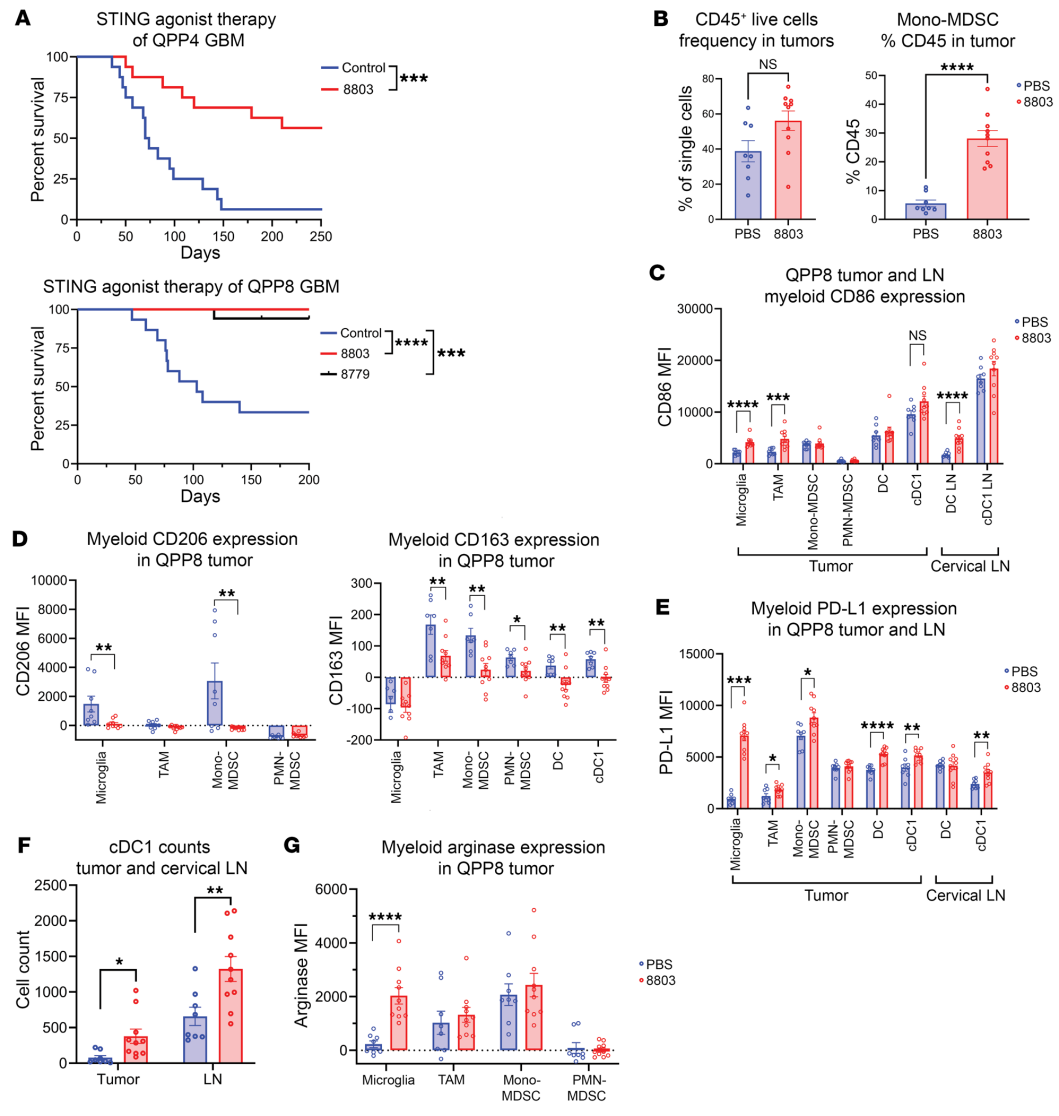
Therapeutic effect of the STING agonist 8803 in immune checkpoint blockade–resistant preclinical models of glioblastoma. (**A**) Treatment of immunocompetent C57BL/6J mice with i.c. implantation of either QPP4 or QPP8 glioma cells. The survival rate of C57BL/6J mice with i.c. implanted QPP4 treated with 8803 (5 μg) on days 14 and 28 (*n* = 16) significantly prolonged survival relative to vehicle control mice (*n* = 16; median survival [MS] = 72 days; log-rank *P* = 0.0003). Similarly, both 8803 and 8879 (*n* = 17; undefined MS) were curative in the QPP8 model (control group: *n* = 15; MS = 103 days) when they were administered on days 14, 21, and 28 after implantation. Agonist 8803 versus vehicle control (log-rank *P* < 0.0001); 8879 versus vehicle control (log-rank *P* = 0.0002). (**B**) Flow cytometric analysis of tumor-infiltrating immune cells using BD LSRFortessa X-30 prototype flow cytometry. QPP8 cells were orthotopically implanted in C57BL/6J mice and then treated with PBS or 5 μg 8803 on days 60 and 67. Tumors were isolated 48 hours after the final treatment and immune cells were collected using a Percoll gradient. The total amount of immune cells was quantified based on all live CD45^+^ cells and specifically on CD11b^+^Ly6C^+^ expression of mono-MDSCs. (**C**) Within the myeloid compartment from the tumor and cervical lymph nodes (LNs), immune cell lineages were identified based on standard surface markers (Supplemental Table 1), and then the mean fluorescence intensity (MFI) was quantified based on treatment. Each dot represents an analyzed tumor or LN. (**D**) Expression of immunosuppressive markers CD206 and CD163 spanning myeloid populations and as a function of treatment. (**E**) Myeloid PD-L1 expression on various immune lineages in tumors and LNs. (**F**) Conventional type 1 DCs (cDC1s) were increased in both tumor and LNs in response to 8803. (**G**) Immunosuppressive arginase expression spanning myeloid populations and as a function of treatment. *P < 0.05; **P < 0.01; ***P < 0.001; ****P < 0.0001 by 2-tailed Student’s *t* test (**B**–**G**).

Others have shown the therapeutic potential of STING activation using the cyclic dinucleotide (CDN) agonist c-di-GMP in preclinical colorectal and melanoma models (28). We have developed 2 highly potent CDN STING agonists, 8803 and 8779, which are 2′,3′-thiophosphate CDN analogs (23). The 8779 agonist has been shown to induce radiographic regression of glioblastoma in dogs (12). A dose escalation of 8803 in mice was performed 10 days after GL261 gliomas were implanted intracranially (i.c.), which showed in vivo therapeutic activity at 5 μg/dose (Supplemental Figure 1; supplemental material available online with this article; https://doi.org/10.1172/JCI175033DS1). GL261 is an immunogenic model of glioblastoma that responds readily to immune checkpoint blockade (29), unlike human glioblastoma. In contrast, the QPP4 and QPP8 glioblastoma models are poorly immunogenic and resist both CTLA-4 and PD-1 blockade (29). Even in these models, we found that 2–3 doses at 5 μg of 8803 could elicit rejection in 56% (QPP4; *P* = 0.0003) to 100% (QPP8; *P* < 0.0001) of animals (Figure 2A). The overwhelming efficacy of STING 8803 agonist therapy in the GL261 and QPP models led us to ask whether a more aggressive, weakly immunogenic model would be less susceptible to this therapy. QPP8 subclones were derived from in vivo–passaged tumors, with the selection of the 2 most aggressive clones (QPP8v and QPP8v2) for implantation studies. Although these aggressive QPP8 derivatives were less sensitive to STING agonist therapy than the parental line, some animals were still cured by 8803 in both settings (Supplemental Figure 2).

### STING agonist 8803 increases myeloid activation in immune checkpoint blockade–refractory glioblastoma.

QPP8 tumors were treated twice, 7 days apart, with 5 μg of the STING agonist 8803 and then isolated and analyzed by flow cytometry 48 hours following the second treatment. With STING agonist therapy, CD45^+^ immune cell frequency increased in the tumors primarily driven by the influx of CD11b^+^Ly6C^+^ MDSCs (Figure 2B). Costimulatory CD86 expression increased in the microglia, tumor-associated macrophages (TAMs), and the DC compartments in the tumor and the DCs residing in the cervical lymph nodes (LNs) (Figure 2C and Supplemental Figure 3). Across the glioblastoma myeloid stroma, we also observed decreased expression of the immunosuppressive markers CD206 and CD163 (Figure 2D). Consistent with the increased IFN release associated with STING agonist therapy, expression of the immune checkpoint ligand PD-L1 also increased across the QPP8 myeloid stroma (Figure 2E). Professional antigen cross-presenting conventional type 1 DCs (cDC1s) increased in frequency, both within the tumor and in the LNs (Figure 2F). Paradoxically, arginase 1 (Arg1) expression, which is upregulated by STAT3 (30), increased across immune lineages in response to STING therapy (Figure 2G).

### STING agonist 8803 functionally enhances both CD8^+^ T and NK cell responses in glioblastoma.

**Figure 3:**
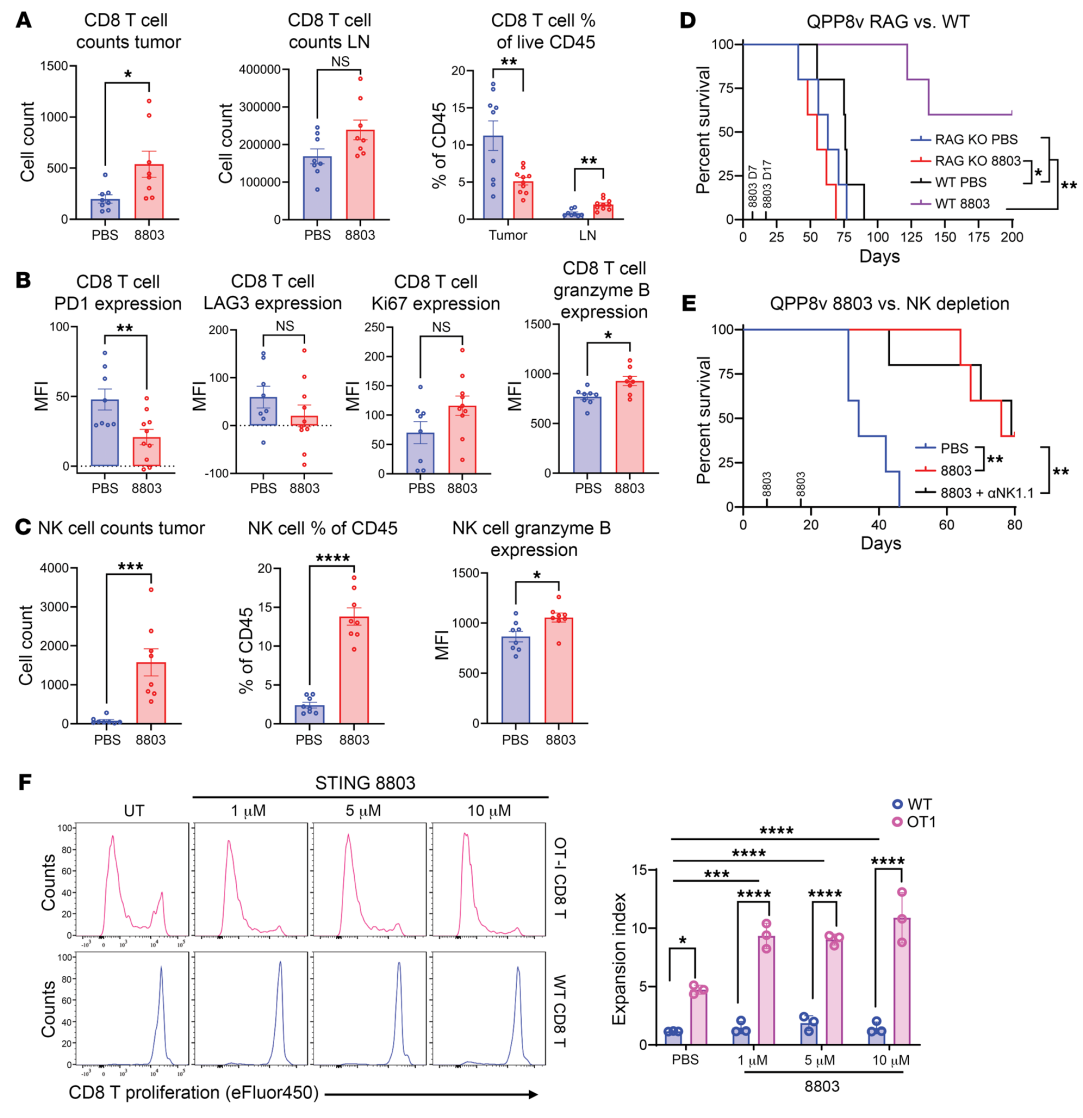
STING agonist 8803 induces immune effector responses within gliomas. Flow cytometric analysis of QPP8-infiltrating immune cells using BD LSRFortessa X-30 prototype flow cytometer. QPP8 cells were orthotopically implanted into C57BL/6J mice and then treated with PBS or 5 μg 8803 on days 60 and 67. Tumors were isolated 48 hours after the final treatment, and immune cells were collected using a Percoll gradient. (**A**) Within the CD8^+^ T cell compartment from the tumor and cervical lymph nodes (LNs), 8803 enhanced the number of infiltrating CD8^+^ T cells. (**B**) CD8^+^ T cell immune exhaustion markers such as PD-1 and LAG-3 were decreased, but proliferation and granzyme B expression increased. (**C**) NK cell infiltration and frequency and granzyme B expression were increased in 8803-treated gliomas. (**D**) The survival rate estimated by the Kaplan-Meier method of RAG^–/–^ versus WT C57BL/6J mice implanted with QPP8v (subclone) and treated with STING agonist 8803 versus PBS. RAG^–/–^ control (PBS): 5 mice (median survival [MS]: 63 days); WT control: 5 mice (MS: 76 days); RAG^–/–^ 8803: 5 mice (MS: 55 days); WT 8803: 5 mice (MS: undefined; 3 long-term survivors). Statistics (log-rank test): RAG^–/–^ control versus WT control *P* = 0.209; RAG^–/–^ control versus RAG^–/–^ 8803 *P* = 0.192; RAG^–/–^ control versus WT 8803 *P* = 0.0018; RAG^–/–^ 8803 versus WT 8803 *P* = 0.0018; RAG^–/–^ 8803 versus WT control *P* = 0.014; WT control versus WT 8803 *P* = 0.0018. (**E**) The survival estimated by the Kaplan-Meier method of C57BL/6J mice implanted with QPP8v and treated with either 8803 or the combination of 8803 + NK1.1 antibody (αNK1.1). Control (PBS): 5 mice (MS: 34 days); 8803: 5 mice (MS: 76 days); 8803 + αNK1.1: 5 mice (MS: 79 days). Statistics (log-rank test): control versus 8803 *P* = 0.0017; control versus 8803 + αNK1.1 *P* = 0.0062; 8803 versus 8803 + αNK1.1 *P* = 0.92. (**F**) Expansion of OT-1 CD8^+^ T cells or WT CD8^+^ T cells collected from spleen and then treated with PBS versus 8803 at 1, 5, and 10 μM. *P < 0.05; **P < 0.01; ***P < 0.001; ****P < 0.0001 by 2-tailed Student’s *t* test (**A**–**C**) or 2-way ANOVA (**F**).

The number of intratumoral CD8^+^ T cells increased with 8803 treatments (Figure 3A), but their frequency within the CD45^+^ population declined due to the higher proportional levels of myeloid expansion. In the cervical LNs, a higher CD8^+^ T cell proportion with STING agonist therapy could be observed. CD8^+^ T cells were less exhausted, with lower PD-1 and LAG-3 expression, had increased cytotoxic potential, and trended toward increased proliferation (Figure 3B). NK cells increased in both number and as a fraction of the glioblastoma immune infiltrates with cytotoxic potential (Figure 3C). To clarify the immune cell population mediating the effector response of 8803, hereafter the QPP8v subclone was implanted into RAG^–/–^ mice, which lack mature B and T cells (see Methods), and responses compared relative to WT C57BL/6J mice (Figure 3D). The absence of T cells in the RAG^–/–^ mice abolished the therapeutic effect of 8803 in comparison with the WT mice (Figure 3D). Additionally, in vivo NK1.1 cell depletion did not ablate the therapeutic activity of 8803 (Figure 3E), indicating that the immune effector population is the T cell. Finally, to ascertain whether 8803 can induce antigen-specific responses, GL261 cells expressing ovalbumin were pretreated with 8803 at 3 doses (1, 5, and 10 μM) and then cocultured with ovalbumin-specific TCR–transgenic OT-1 CD8^+^ T cells or WT control T cells (Figure 3F). Antigen-specific T cell expansion was detected at 1 μM and was not dose-dependent.

### Spatial STING expression and downstream activation in glioblastoma.

**Figure 4:**
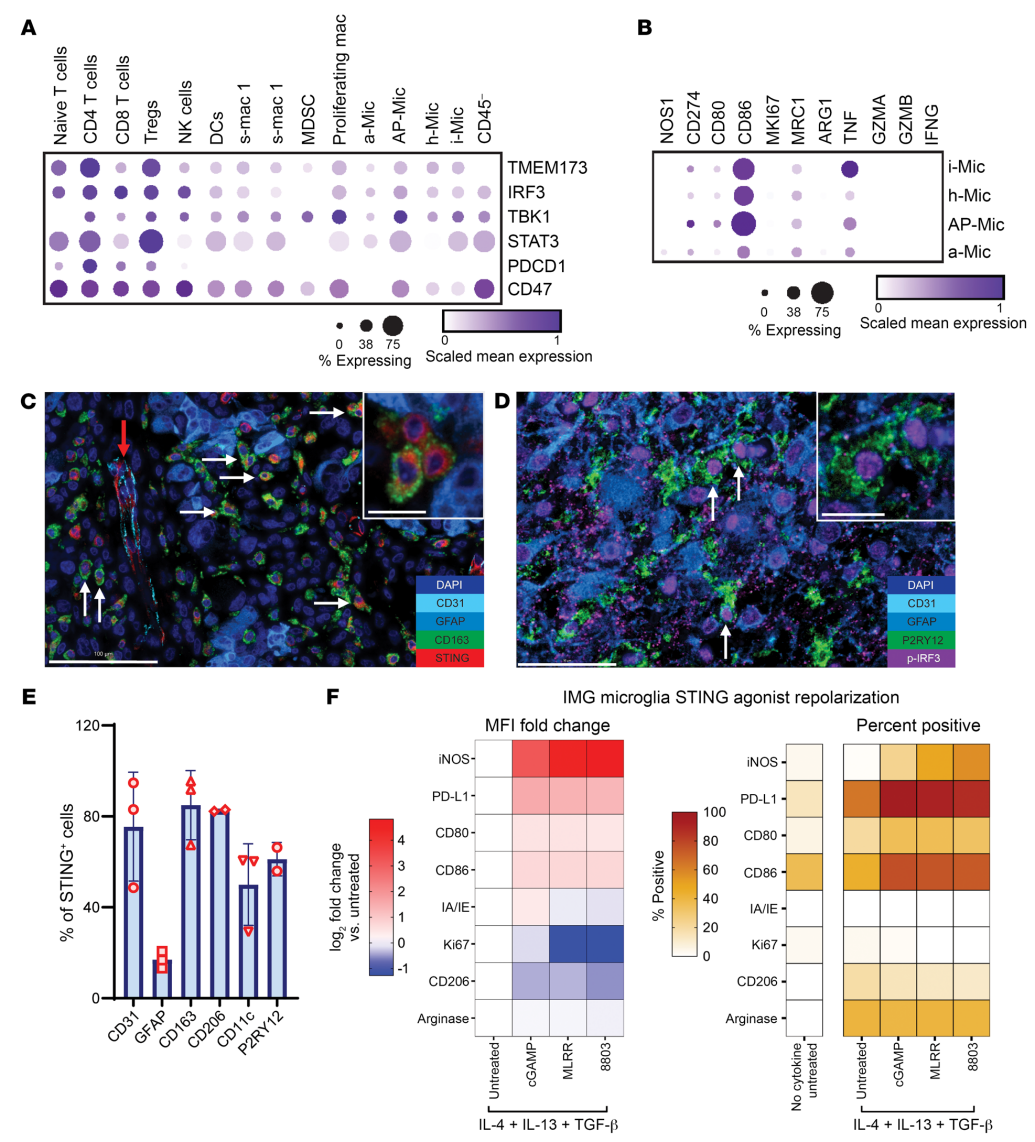
STING expression within human glioblastoma microglia and reprogramming with 8803. (**A**) Dot plot showing key gene expression from scRNA-seq of 44 tumor fragments representing 18 glioma patients, including low-grade gliomas (*n* = 2), newly diagnosed glioblastoma (*n* = 11), and recurrent glioblastoma (*n* = 5) analyzed from Abdelfattah et al. (56). Bubble size corresponds to the percentage of cells expressing a gene marker; colors indicate scaled mean expression. (**B**) Dot plot of selected gene expression within microglia subtypes. CD45^–^ cells include both endothelial and tumor cells. (**B**) Immune effector functions of microglia subtypes. (**C**) Representative multiplexed sequential immunofluorescence (SeqIF) imaging of human glioblastoma demonstrating the expression of STING in CD163^+^ macrophages denoted by white arrows in proximity to the CD31 tumor vasculature (red arrow). Scale bar: 100 μm. A higher magnification image of STING^+^ CD163^+^ cells is represented at the upper right quadrant (scale bar: 20 μm). (**D**) Representative multiplexed SeqIF imaging of human glioblastoma, demonstrating the expression of p-IRF3 (downstream activation of STING pathway) in P2RY12^+^ microglia denoted by the white arrows. Scale bar: 50 μm. A higher magnification image of p-IRF3^+^P2RY12^+^ cells is represented at the upper right quadrant (scale bar: 20 μm). (**E**) Quantification plot showing the percentages of STING expression in the different cell populations within the human glioblastoma TME. (**F**) IL-4–, IL-13–, and TGF-β–polarized murine IMG microglia were treated for 48 hours with STING agonists (10 μg/mL), with increasing potency from cGAMP to MLRR-S2-CDA to 8803, and profiled based on various markers. These were quantified based on MFI fold change or percentage of cells that are positive and then presented as a heatmap.

To gain further insights into the immunological role of STING in glioblastoma, we profiled single-cell RNA-seq (scRNA-seq) data sets for the mRNA expression of STING, IRF3, Tbk-1, STAT3, PD-1, and CD47 based on annotated immune cell lineages. CD47 was included in this analysis since it has been used as a targeting moiety for STING (31). In contrast with the T cell–confined expression of PD-1, STING, IRF3, and Tbk-1 were expressed in both the T cell and myeloid immune compartments, including various subsets of microglia (Figure 4A). Glioblastoma microglia were subtyped into the following: inflammatory microglia (i-Mic) that express *TNF* and *IL1B*; hemostatic (h-Mic) that express *CST3*; antigen-presenting (AP-Mic) that express both microglia and macrophage markers such as *CX3CR1*, *CD86*, *IFNGR1*, *TGFB1*, and *B2M*; and activated microglia (a-Mic) that display activation markers. At baseline, although these microglia subsets do share some immunological features, STAT3 is expressed mainly in the AP-Mic and the i-Mic (Figure 4A), whereas the i-Mic express more TNF-α (Figure 4B). When profiled at the protein level using multiplexed SeqIF staining, the expression of p-STAT3, STING, and the activated STING pathway biomarker p-IRF3 converge on the endothelial compartment, and the myeloid populations, such as CD163^+^ and CD206^+^ macrophages, CD11c^+^ antigen-presenting cells (Figure 4, C and E, Supplemental Figure 4C, and Supplemental Video 1), and microglia (Figure 4, D and E) within human glioblastoma. When p-IRF3 expression was analyzed from surgical specimens that included adjacent infiltrating brain (*n* = 2), P2RY12^+^ microglia were the most abundant immune cell in the adjacent infiltrating brain (Supplemental Figure 4D), and they express p-IRF3 (Figure 4D). Although STAT3 expression could be detected in the glioma cells, the STING pathway activation markers were mostly found in the immune cell populations such as CD11c^+^ antigen-presenting cells, CD163^+^ macrophages, and P2RY12^+^ microglia at the edge of the tumor. The expression of PD-1 and PD-L1 was rare, as we previously described (32), and was mostly aligned to the expression on T cells and CD11c^+^ antigen-presenting cells, respectively (33).

### Microglia polarized to immunosuppression undergo proinflammatory conversion in response to STING activation.

Immunosuppressive microglia are present within the glioblastoma TME, yet their response to STING activation has not been previously described. We used a combination of IL-4 and IL-13 with and without TGF-β to drive BV2 or IMG microglial cell lines to an immunosuppressive phenotype (Supplemental Figure 4, A and B) that mimics their glioblastoma-resident counterparts. Next, we treated these microglia with STING agonists of increasing potency (2′,3′-cGAMP < MLRR-S2-CDA < 8803) (Figure 4F). In each case, STING activation led to increases in expression of the T cell costimulatory molecules CD80 and CD86 and a decrease in the tumor-supportive, immunosuppressive marker CD206 (Figure 4F and Supplemental Figure 4, A and B). While the M1 myeloid marker inducible nitric oxide synthase (iNOS) also increased, so did the expression of the T cell checkpoint ligand PD-L1. Like prior studies of STING effects on MDSCs and TAMs (34, 35), IMG microglia proliferation (Ki67) was almost completely halted by the more potent STING agonists; however, BV2 proliferation increased in this setting (Supplemental Figure 4, A and B). While these data support the capacity of a synthetic STING agonist to mediate the proinflammatory conversion of tumor microglia, they also suggest that concomitant use of PD-1 blockade might be necessary.

### STING agonist 8803 demonstrates therapeutic activity in epigenetically silenced glioblastoma and does not induce CNS autoimmunity.

**Figure 5:**
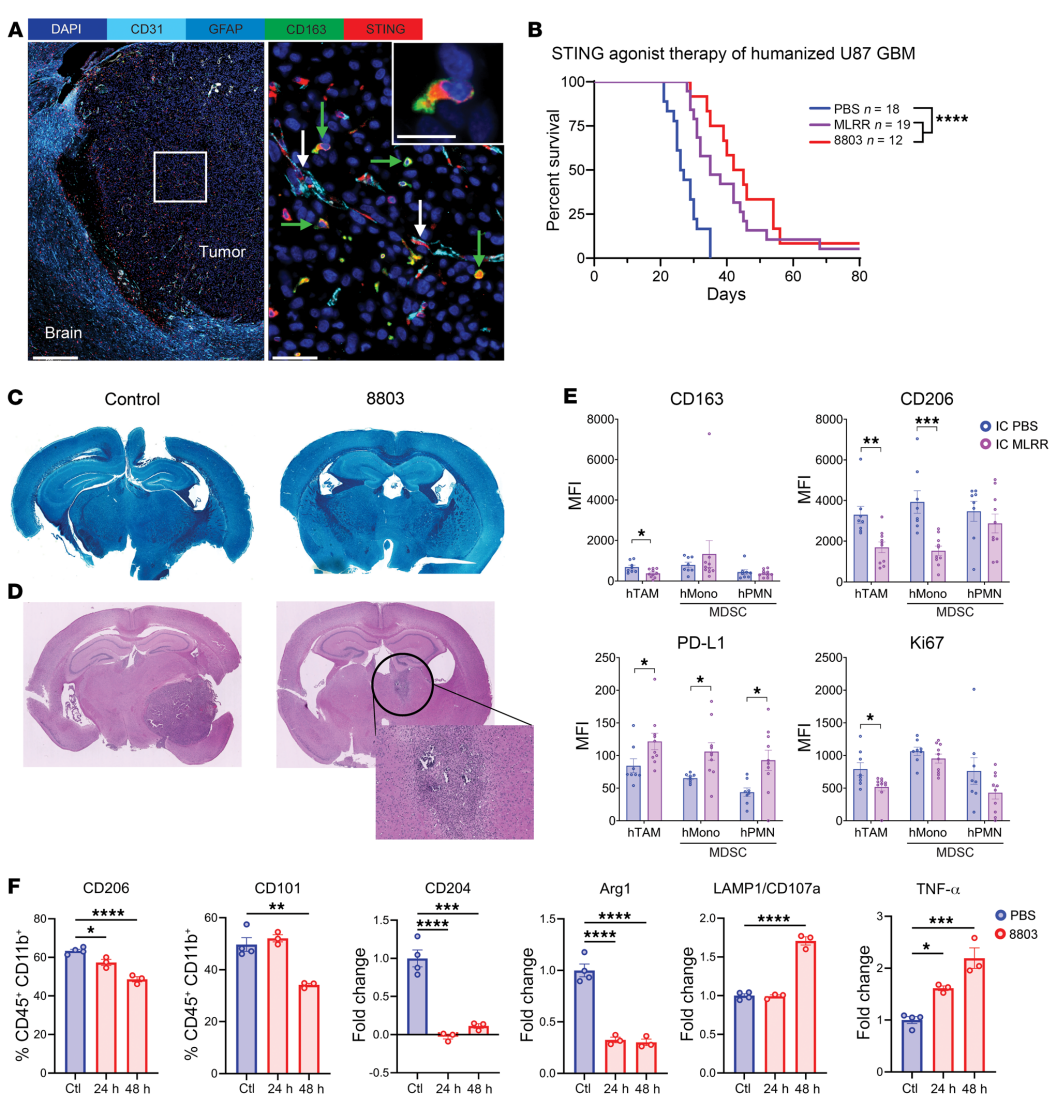
Therapeutic effect of STING agonist 8803 in humanized glioma mouse model recapitulating human glioblastoma. (**A**) Multiplexed sequential immunofluorescence images of the STING expression at baseline in humanized mouse brain implanted with U87 glioma cells and collected at endpoint. The right image represents a high magnification of the white box drawn in the left image. The white arrows highlight CD31^+^STING^+^ vessels and green arrows highlight the CD163^+^STING^+^ macrophages. DAPI (dark blue), GFAP (light blue), CD31 (cyan blue), STING (red), CD163 (green). Scale bars: 500 μm (left panel) and 50 μm (right panel). A higher magnification image of a CD163^+^STING^+^ cell is represented at the upper right quadrant of the right image (scale bar: 20 μm). (**B**) Humanized mice that underwent i.c. implantation of 112.5 × 10^3^ (survival) or 90 × 10^3^ (immune infiltrate analysis) human U87 glioma cells treated with PBS (*n* = 18), the moderately potent STING agonist MLRR-S2-CDA (*n* = 19), or 8803 (*n* = 12) on days 5, 10, and 15. The survival rate of the humanized mice was estimated by the Kaplan-Meier method. Control: median survival (MS): 26.5 days, MLRR-S2-CDA MS: 35 days, 8803 MS: 43.5 days. Statistics: control versus MLRR-S2-CDA log-rank ****P < 0.0001; control versus 8803 log-rank ****P < 0.0001. (**C**) Luxol Fast Blue demonstrating uniform staining without evidence of clearance that would be reflective of demyelination in the CNS in either the control or 8803-treated brains (×1.5 magnification). (**D**) Representative hematoxylin and eosin–stained coronal sections of mice at the survival endpoint demonstrating persistent glioma after treatment with 8803. Original magnification, ×1.25 (left and middle images) and ×10 (bottom right image). (**E**) Ex vivo Flow cytometric analysis of U87-infiltrating human immune cells using BD LSRFortessa X-30 prototype flow cytometer. MFI, mean fluorescence intensity; h, human; TAM, tumor-associated macrophage; Mono, monocyte; PMN, peripheral mononuclear cell; MDSC, myeloid-derived suppressor cell. (**F**) WT C57BL/6J mouse bone marrow–derived macrophages pretreated with IL-4 for 48 hours followed by STING agonist 8803 for the indicated times (24 hours and 48 hours). The markers were assessed via Cytek Aurora flow cytometer and the CD45^+^CD11b^+^ population was analyzed for the indicated markers. *P < 0.05; **P < 0.01; ***P < 0.001; ****P < 0.0001 by 2-tailed Student’s *t* test (**E**–**F**).

We have previously shown that 8803 exerts therapeutic activity in T cell–deficient pancreatic cancer (15). Because STING was shown to be epigenetically silenced in human gliomas (36), STING expression on human and murine glioma cells was analyzed (Supplemental Figure 5A). The cell lines U87, GL261, and CT-2A were selected for this analysis because they may recapitulate the human glioblastoma biology of epigenetic silencing of STING. Although there are significant genetic and immunologic similarities between the QPP glioblastoma lines and human glioblastoma, we sought to evaluate the effects of STING agonist therapy in CD34^+^ stem cell–humanized huNOG-EXL mice. Effectively, in the U87 tumors implanted in the humanized mice, STING expression at the protein level was observed on the endothelium (CD31^+^, white arrows) and within the CD163^+^ macrophages (green arrows) within the TME (Figure 5A). Both 8803 and another prototype synthetic STING agonist, MLRR-S2-CDA, significantly extended survival of U87 tumor–bearing animals (both *P* < 0.0001), confirming the efficacy of this approach in a system with human lymphocytes and myeloid cells (Figure 5B). To evaluate whether this treatment induces an inflammatory response directed against the CNS, the mice were evaluated daily. No neurological symptoms were noted. When the mice succumbed to the tumor, the neuroaxis was stained for myelin with Luxol Fast Blue, and hematoxylin and eosin. On histologic examination by a board-certified neuropathologist, there was no evidence of autoimmune demyelinating encephalomyelitis (Figure 5, C and D). Additionally, neither perivascular lymphocytic infiltrates nor vascular fibrosis were seen, which otherwise might have suggested induction of low-grade autoimmunity. To verify that the absence of induced autoimmunity was not simply a function of the model analyzed, both C57BL/6J control and 8803-treated mice were analyzed similarly and likewise showed no evidence of demyelination. An invasion assay was conducted on glioma tumor cells, indicating that 8803 does not enhance the underlying invasive properties of these cells (Supplemental Figure 5B). Cumulatively, these data indicate that 8803 is exerting an immunological and therapeutic effect in a glioma model in which STING has been deactivated in the tumor cell. Although the STING-treated mice succumbed to the disease, the tumors were smaller than those of controls (Figure 5D).

### STING agonists reverse the myeloid immunosuppressive phenotype.

Ex vivo analysis of the human myeloid population from the humanized U87 model demonstrated that the STING agonist MLRR-S2-CDA reduced the expression of CD163 and CD206 on various populations of human MDSCs and TAMs (Figure 5E). Similarly, this reversal of immunosuppression was also detected with 8803-treated bone marrow–derived macrophages polarized with IL-4 to an immunosuppressive M2 phenotype. Agonist 8803 markedly decreased immunosuppressive markers such as CD206, CD101, CD204, and Arg1, while increasing prophagocytic (LAMP1) and proinflammatory TNF-α over time (Figure 5F).

### WP1066-mediated STAT3 pathway blockade with 8803 does not enhance survival in preclinical models of glioblastoma.

**Figure 6:**
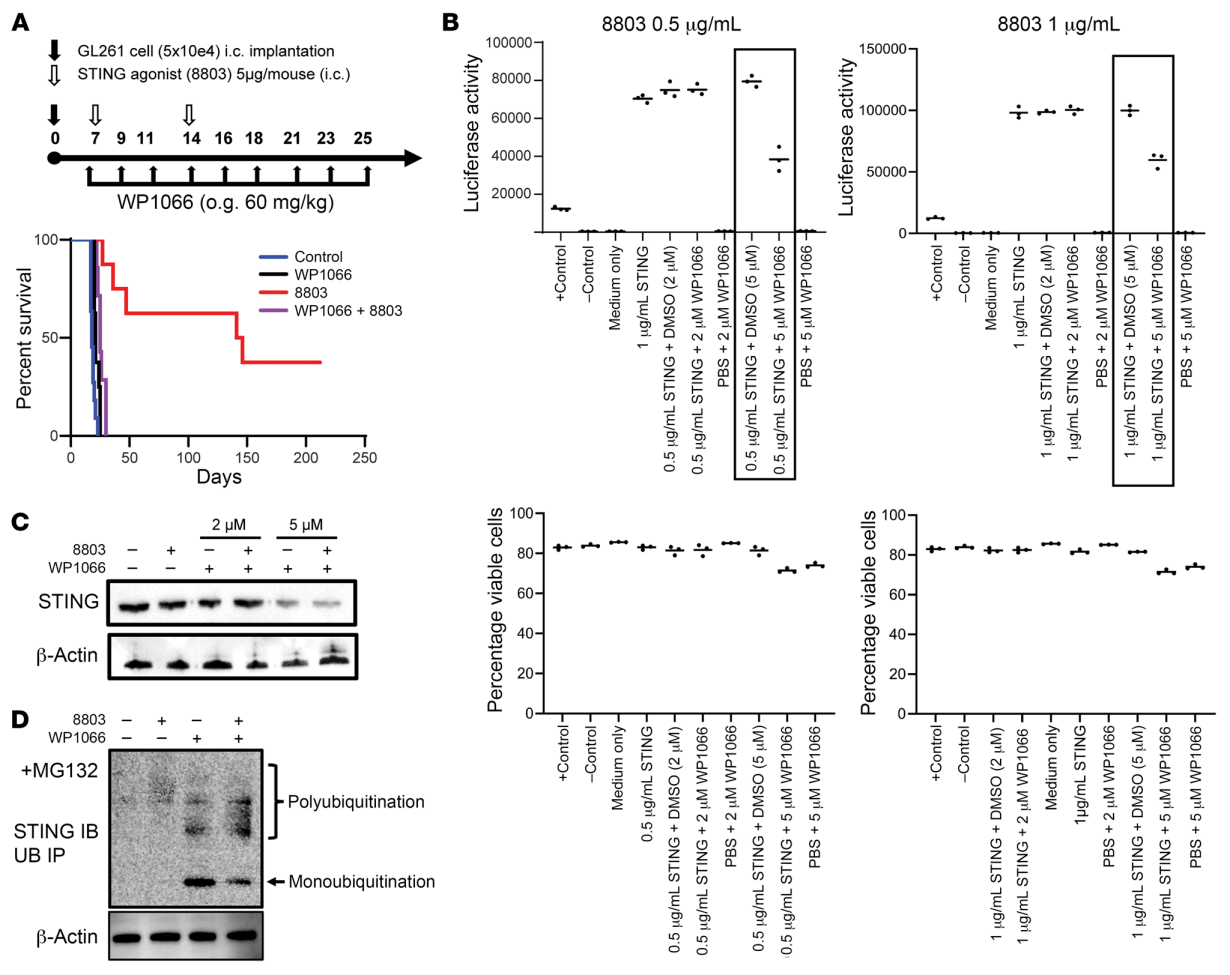
The STAT3 inhibitor, WP1066, in combination with STING in the GL261 murine glioma model. (**A**) Schema of the treatment of immunocompetent mice that underwent i.c. implantation of GL261 glioma cells. Seven days after GL261 implantation, mice were treated with WP1066 (60 mg/kg) by oral gavage (o.g.) 3 times per week (M/W/F) for 3 weeks. On day 7, mice were treated intratumorally with the STING agonist 8803 (5 μg). The survival rate of C57BL/6J mice was estimated by the Kaplan-Meier method. Control: 11 mice (median survival [MS]: 18 days), WP1066: 8 mice (MS: 21 days), 8803: 8 mice (MS: 143.5 days); 4 long-term survivor), WP1066 + 8803 agonist: 7 mice (MS: 25 days). Statistics (log-rank test): control versus WP1066 *P* = 0.0047; control versus 8803 *P* < 0.0001; control versus WP1066 + 8803 *P* = 0.0002; WP1066 versus WP1066 + 8803 *P* = 0.02; 8803 versus WP1066 + 8803 *P* = 0.0005; 8803 versus WP1066 *P* < 0.0001. (**B**) In vitro luciferase expression assay for the induction of IFN responses. Various concentrations of the STING agonist 8803 were used to induce the luciferase expression in the top panels. A physiological (2 μM) and a high dose (5 μM) of WP1066 was used in combination. Direct cellular cytotoxicity was measured during the above experimental conditions (lower panels). At a WP1066 concentration of 5 μM, there was a decrease in IFN activity, which was not attributed to cell viability. (**C**) WP1066 decreases STING protein expression in THP-1 cells in a dose-dependent manner. THP-1 cells were treated with the indicated concentrations of WP1066. Cells were collected and analyzed by Western blotting. (**D**) Ubiquitination assay of STING in THP-1 cells indicates that WP1066 induces STING ubiquitination in a dose-dependent manner. MG132, proteasome inhibitor used in all conditionsl; IB, immunoblotting; UB, ubiquitination blotting; IP, immunoprecipitation.

A key mechanistic hub of glioblastoma-mediated immunosuppression, especially in myeloid cells, is the p-STAT3 pathway (37). Since the blood-brain-barrier penetrant small molecule STAT3 inhibitor, WP1066, is entering phase II clinical trials (ClinicalTrials.gov NCT05879250), we wanted to determine whether there is a benefit to combining WP1066 with 8803. To evaluate the activity of WP1066 in a glioma model that is highly resistant to the effects of WP1066, we selected the GL261 model (22). C57BL/6J mice bearing i.c. GL261 tumors were treated with 8803 and/or WP1066. The mice were randomized to receive (i) WP1066 (60 mg/kg for 3 weeks on Monday, Wednesday, and Friday); (ii) STING agonist 8803 (5 μg/mouse); (iii) WP1066 + 8803; and (iv) control. WP1066 treatment was started on day 7 after tumor implantation, and 8803 was administered on days 7 and 14 (Figure 6A). Long-term durable survival was observed only with monotherapeutic 8803 (Figure 6A). More specifically, the control mice had a median survival time of 18 days, WP1066-treated mice had a median survival time of 21 days, 8803-treated mice had a median survival time of 143.5 days (*P <* 0.05 vs. control), and 8803 + WP1066–treated mice had a median survival time of 25 days. Forty percent of mice treated with 8803 were long-term survivors (>150 days after tumor implantation).

### High doses of WP1066 inhibit STING downstream IRF3 induction.

To clarify why the WP1066 compound might ablate the therapeutic effect of the STING agonist, THP-1 cells, which have stable integration of an inducible reporter construct that enabled the study of the activation of the IRF3 pathway, were used. Luciferase is controlled by the IFN-stimulated gene 54 (ISG54) promoter. If STING is active, then a luciferase signal is detected. Two different concentrations of the STING agonist were tested (0.5 and 1 μg/mL) in combination with a physiological (2 μM) and superphysiological concentration (5 μM) of WP1066. WP1066 at 5 μM decreased luciferase expression of IFN response genes induced by STING (Figure 6B). Cell viability was not affected at 2 μM WP1066, but at 5 μM, viability was slightly reduced by 12%–13%, with no significant difference in comparison to the other groups (*P* > 0.05). Cumulatively, these data indicate that WP1066 is not ablating STING activation at the promoter site, instead suggesting that a posttranslational process may be responsible.

### WP1066 induces ubiquitination of STING.

Many of the key proteins and signaling pathways such as HIF-1α, c-Myc, STING, and cancer stemness that WP1066 modulates converge on mechanistic control by ubiquitination (38). WP1066 is modified from AG490, which is a natural tyrphostin that can induce cellular apoptosis through the process of inhibiting deubiquitinase activity (39). Since the combination therapy of 8803 and WP1066 did not improve survival compared to monotherapeutic 8803, we hypothesized that WP1066 might promote ubiquitination of STING. To test this possibility, the effect of WP1066 on the STING protein expression level was analyzed at various concentrations of WP1066. THP-1 cells were used for monitoring the activity of IFN-γ–induced signal transduction pathways. At lower concentrations of WP1066, there was no loss of protein expression of STING; however, at higher concentrations, the expression of STING was lost (Figure 6C). This decrease in STING protein was associated with polyubiquitination after WP1066 treatment (Figure 6D).

### Dose and schedule modifications of WP1066 restore the therapeutic activity of 8803 in vivo.

**Figure 7:**
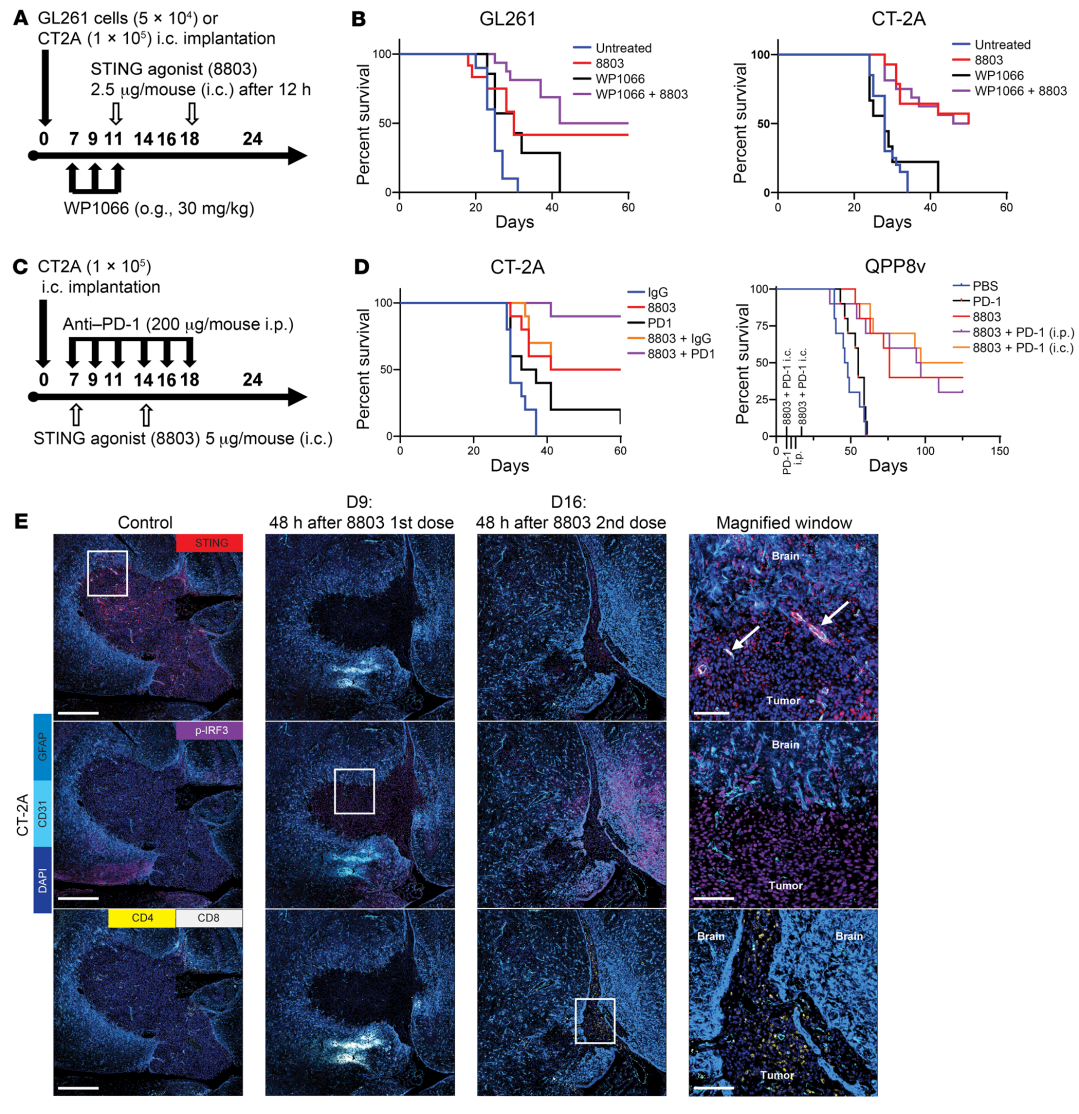
Dose and schedule adjustments for combinatorial WP1066 with 8803: combination with anti–PD-1. (**A**) Schema of the treatment of immunocompetent mice that underwent i.c. implantation of either GL261 or CT-2A glioma cells. (**B**) The survival rate estimated by the Kaplan-Meier method of C57BL/6J mice implanted with GL261 and CT-2A. For the GL261 survival: control: 10 mice (median survival [MS]: 25 days), WP1066: 10 mice (MS: 30 days), 8803: 14 mice (MS: 30 days; 5 long-term survivors), WP1066 + 8803: 16 mice (MS: 71 days; 8 long-term survivors). Statistics (log-rank test): control versus WP1066 *P* = 0.04; control versus 8803 *P* = 0.01; control versus WP1066 + 8803 *P* < 0.0001; WP1066 versus WP1066 + 8803 *P* = 0.006; 8803 versus WP1066 + 8803 *P* = 0.32. For the CT-2A survival: control: 10 mice (MS: 28 days), WP1066: 9 mice (MS: 28 days), 8803: 14 mice (MS: 50 days; 7 long-term survivors), WP1066 + 8803: 16 mice (MS: 48 days; 8 long-term survivors). Statistics (log-rank test): control versus WP1066 *P* = 0.57; control versus 8803 *P* < 0.0001; control versus WP1066 + 8803 *P* < 0.0001; WP1066 versus WP1066 + 8803 *P* < 0.001; 8803 versus WP1066 + 8803 *P* = 0.95. (**C**) Schema of the treatment of immunocompetent mice that underwent i.c. implantation of either CT-2A or QPP8v (subclone) glioma cells. QPP8v tumors received 8803 (5 μg/mouse i.c. on days 7 and 17), anti–PD-1 (25 μg i.c. on days 7 and 17), and vehicle control (PBS) or anti–PD-1 (250 μg i.p. on days 7, 10, and 13) in single and combination therapies. (**D**) The survival rate estimated by the Kaplan-Meier method of C57BL/6J mice implanted with CT-2A and QPP8. For CT-2A: IgG: 10 mice (MS: 30 days); anti–PD-1: 10 mice (MS: 35 days), 8803: 10 mice (MS: 56 days, 4 long-term survivors); 8803 + anti–PD-1: 10 mice (MS: undefined, 8 long-term survivors); 8803 + IgG: 10 mice (MS: 65.5 days, 5 long-term survivors). Statistics (log-rank test): IgG versus anti–PD-1 *P* = 0.04; IgG versus 8803 *P* < 0.01; IgG versus 8803 + anti–PD-1 *P* < 0.0001; anti–PD-1 versus 8803 + anti–PD-1 *P* < 0.001; 8803 versus 8803 + anti–PD-1 *P* = 0.04; 8803 + IgG versus 8803 + anti–PD-1 *P* = 0.12. For QPP8: PBS: 10 mice (MS: 47 days); anti–PD-1: 10 mice (MS: 55 days); 8803: 10 mice (MS: 76 days, 4 long-term survivors); 8803 + anti–PD-1 (i.p.): 10 mice (MS: 95.5 days, 3 long-term survivors); 8803 + anti–PD-1 (i.c.): 10 mice (MS: 111 days, 5 long-term survivors). Statistics (log-rank test): PBS versus anti–PD-1 *P* = 0.164; PBS versus 8803 *P* < 0.0001; PBS versus 8803 + anti–PD-1 (i.p.) *P* = 0.0006; PBS versus 8803 + anti–PD-1 (i.c.) *P* < 0.0001; anti–PD-1 versus 8803 *P* < 0.0001; anti–PD-1 versus 8803 + anti–PD-1 (i.p.) *P* = 0.002; anti–PD-1 versus 8803 + anti–PD-1 (i.c.) *P* < 0.0001; 8803 versus 8803 + anti–PD-1 (i.c.) *P* = 0.57; 8803 versus 8803 + anti–PD-1 (i.p.) *P* = 0.85. (**E**) Multiplexed sequential immunofluorescence images of untreated CT-2A gliomas (*n* = 3). Forty-eight hours after either the first (day 9) or second dose (day 16) of 8803, animals were euthanized and the brains were imaged for the following (*n* = 3/group): DAPI (dark blue), GFAP (light blue), CD31 (cyan blue), STING (red), p-IRF3 (pink), CD4^+^ (yellow), and CD8^+^ (white) T cells. White boxes outline the portion shown at higher magnification in the right column of images. Scale bars: 500 μm (left 3 columns) and 50 μm (right column).

The dose of WP1066 was reduced and the administration of the STING agonist was delayed to try to prevent the induction of ubiquitination. The C57BL/6J mice bearing i.c. GL261 tumors were randomized to receive (i) WP1066 (30 mg/kg for 3 weeks on Monday, Wednesday, and Friday); (ii) STING agonist 8803 (2 μg/mouse); (iii) WP1066 + 8803; and (iv) control. WP1066 treatment was administered on days 7, 9, and 11 after tumor implantation, and the STING agonist was administered on days 11 and 18 (Figure 7A). The untreated mice had a median survival time of 25 days, WP1066-treated mice had a median survival time of 25 days, STING-treated mice had a median survival time of 29 days (*P* = 0.27 vs. control), and STING + WP1066–treated mice had a median survival time of 71 days. Fifty percent of mice treated with the combination were long-term survivors (>150 days after tumor implantation), which was statistically significant relative to controls (*P* < 0.024) and WP1066 monotherapy (*P* = 0.024), but not relative to monotherapeutic STING agonist (*P* = 0.68) (Figure 7B). The same dose and schedule were evaluated in a second immunocompetent murine model of GBM, CT-2A, and no additive benefit of the combination of WP1066 and 8803 was detected (Figure 7B).

### STING agonist 8803 demonstrates synergy with anti–PD-1.

PD-1 is expressed on macrophages in the glioma microenvironment, and anti–PD-1 antibodies can induce proinflammatory M1 responses (33). Since a STING agonist would stimulate a proinflammatory response, the modulation of the PD-1 pathway may further potentiate the therapeutic effect of STING. As such, we evaluated synergy in the CT-2A and QPP8v immunocompetent murine glioma models. C57BL/6J mice bearing i.c. CT-2A or QPP8v tumors were treated with the STING agonist 8803 and/or anti–PD-1. The CT-2A mice were randomized to receive (i) IgG control (200 μg/mouse administered i.p. 3 times per week for 2 weeks); (ii) anti–PD-1 (200 μg/mouse administered i.p. 3 times per week for 2 weeks); (iii) STING agonist 8803 (5 μg/mouse administered i.c. once per week for 2 weeks); (iv) 8803 + IgG control; (v) 8803 + anti–PD-1; and (vi) untreated. The QPP8v mice received (i) i.c. vehicle control; (ii) 8803 (5 μg/mouse i.c. on days 7 and 17 after implantation); (iii) 8803 + anti–PD-1 (25 μg/mouse i.c. on days 7 and 17); or (iv) 8803 + vehicle control or anti–PD-1 (250 μg i.p. on days 7, 10, and 13) (Figure 7C). Long-term durable survival was observed with both monotherapeutic STING agonist and in combination with anti–PD-1 (Figure 7D). More specifically, in the CT-2A glioma model, the control IgG mice had a median survival time of 30 days, 8803-treated mice had a median survival time of 49 days with 50% long-term survivors when the experiment was terminated at 80 days, anti–PD-1–treated mice had a median survival time of 35 days, and the combination had an undefined median survival in which 90% of mice treated with the combination therapy were long-term survivors, which was statistically significant relative to controls and to anti–PD-1 and STING agonist monotherapies (*P* < 0.05). In the QPP8v model, there was no significant treatment benefit of the combination therapy of anti–PD-1 with 8803, including with anti–PD-1 being delivered directly into the glioma (Figure 7D), suggesting that this combination would likely only be of benefit in scenarios predisposed to respond to immune checkpoint inhibitors. To visualize TME features that would be reflective of response, the CT-2A model was treated on day 7 and during the therapeutic window, the activation of the STING pathway (i.e., p-IRF3) and the immune infiltration were analyzed with multiplexed SeqIF imaging. In the CT-2A model, STING expression was present along the tumor vasculature, but also within the tumor cells, albeit at much less intensity. Forty-eight hours after the first dose of 8803 therapy, there was shrinkage of the tumor size in comparison with control (Figure 7E and Supplemental Figure 5C). Notably, the p-IRF3 expression was diffusely present. On day 16, 48 hours after the second dose of STING, both CD4^+^ and CD8^+^ T cells could be found in the region of the prior tumor (Figure 7E).

## Discussion

In multiple murine glioma models, therapeutic activity was observed with 8803, providing a strong rationale for translational implementation. These models included a humanized mouse model in which the STING pathway has been epigenetically silenced in the glioblastoma cells, recapitulating the biology of human patients. This immunological elimination of glioblastoma is mediated by the orchestrated and cooperative interaction between both the innate and adaptive immune components. The observations that STING agonists upregulate costimulation and downmodulate M2 polarization while enhancing T and NK cell proliferation, effector responses, and reducing exhaustion from the glioblastoma TME are consistent with the findings of STING agonists described for other malignancies. Our study reports several observations, including that the expression of STING at the tumor-endothelial junction under baseline untreated conditions is inadequate for facilitating immune cell infiltration and propagation throughout the human glioblastoma TME. The spatial confinement of the T cells in the perivascular space is possibly through the regulation of CXCL12. Our data implicate STING pathway expression within endothelial cells as the instigator of T cell inflammatory responses in the glioblastoma TME and is likely one of the reasons behind the limited effects of T cell–targeted therapies.

In the current study, 8803 was directly administered into the glioblastoma, overcoming the limitations of off-target effects, pharmacokinetic clearance, and the blood-brain barrier. Formulation in a hydrogel for sustained delivery or targeting to myeloid cells using nanotherapeutics is currently under development. Because the mechanism of action for 8803 is through the immune system and is not a direct cytotoxic agent, the entire TME does not need to be exposed to this agent for it to mediate an effect. Despite 8803 being directly injected into the glioblastomas within the brain, this was well tolerated, and no neurological toxicities were observed. When the CNS was evaluated with Luxol Fast Blue, there was no evidence of induced autoimmunity. This may be because the STING pathway is not activated/present within the normal brain vasculature.

The most profound therapeutic effect of 8803 was found in the QPP8 murine model. This glioblastoma cell line does not have epigenetic silencing of the STING pathway, which would suggest that strategies that demethylate STING in the glioblastoma cell population may have synergy with STING agonists. As there are differences in the methylation of the STING promoter between humans and mice, the preclinical murine studies may be an overestimate of the therapeutic impact of STING agonists. In the case of the humanized murine model with U87, the noted increase in survival could be mediated through tissue rejection through MHC incompatibility, reflecting a tumor rejection paradigm. However, we have previously shown that a closely related 8803 analog, 8779, induces radiographic regression in spontaneously arising glioblastomas in dogs whose glioblastomas are associated with a high abundance of macrophages within the TME (12). The promoter methylation status of STING in canine glioblastoma is unknown. Cumulatively, our study and others indicate that even in malignancies that lack STING expression such as glioblastoma, the myeloid and endothelial stroma may mediate the in vivo responses.

Human microglia have been shown to express the cGAS/STING pathway (40). The role of STING in microglia is emerging as an important mechanism in neurodegenerative disorders such as Alzheimer’s (41, 42) and Parkinson’s (43). In preclinical stroke models, METTL14 is upregulated in microglia/macrophages, which then enhances the expression of KAT3B by promoting m6A modification of KAT3B mRNA. KAT3B increases STING expression by enhancing H3K27ac in the STING promoter. METTL14 promotes M1 polarization and the inflammasome axis by KAT3B/STING signaling after stroke (44). Targeting the STAT3 pathway to inhibit STING activation has been shown to improve neuronal senescence after ischemic stroke (45). Another group has shown that STING activity is increased in aged microglia (46), but we now show that STING expression is a function of proximity to the malignancy. The induction of neurodegeneration requires chronic, constitutively active STING in preclinical models (47). Whether long-term survivors of glioblastoma who have been treated with STING agonists ultimately develop problems with neurodegenerative disorders will need to be assessed in the context of clinical trials that include cognitive testing. Ongoing IND-enabling studies for 8803 will evaluate the immunological phenotype of CNS immune cells, including microglia, as a function of distance from the site of administration, and the kinetics of STING pathway activation within the healthy brain of preclinical models.

There is an immunological rationale for combining STING activation with STAT3 pathway modulation (24). When tested preclinically, we were surprised that there was no additive or synergist effect. The Federal Drug Administration (FDA) typically uses the maximum tolerated dose (MTD) to guide dosage recommendations in oncology. This strategy theoretically provides the highest possible therapeutic response while minimizing toxicity. This approach is a common default for dose selection, but biological therapeutics such as monoclonal antibodies are highly targeted and may not necessarily benefit from a “more is better” strategy. The FDA Project Optimus campaign encourages investigators to establish a minimum effective dose (MED) and effective range of dosing with the potential of minimizing toxicity (48). Our analysis demonstrates that higher concentrations of the STAT3 inhibitor WP1066 evaluated in phase I studies (21) induce ubiquitination that precluded its use with some combinatorial approaches. Our previous study demonstrated that this high concentration of WP1066 (60 mg/kg) can be used with radiation (22), whose mechanism of activity is likely unaffected by ubiquitination. Even with dose modifications and schedule changes, this combination needs to be deprioritized for consideration in clinical trials with these specific agents. Alternative strategies involving other STAT3 modulators could be considered. Our group has developed a dual STAT3- and ubiquitination-blocking small molecule designated WP1732 that is currently undergoing preclinical testing to be described separately in the future.

Other strategies for activating the STING pathway include the conjugation of a CpG oligodeoxynucleotide to a targeting moiety. One example is a STING agonist–targeting CD47 antibody that could induce M1 polarization in TAMs, reduce immunosuppression, and inhibit orthotopic glioblastoma by phagocytosis of macrophages and microglia (31). Because there is expression of CD47 on the tumor cells in the CD45^–^ compartment, a substantial fraction of this drug would be shuttled to the tumor cell in which the STING pathway is shut off secondary to STING promoter methylation. A STING agonist strategy affecting a broad range of tumor, immune, and non-immune cells may have a greater therapeutic impact. The high expression of the STING pathway within the myeloid compartment throughout the glioblastoma TME indicates that whether 8803 is injected into the myeloid-enriched tumor itself or the surgical bed infiltrated with microglia, this pathway can be therapeutically modulated. Therapeutic activity can be further enhanced when used in combination with anti–PD-1 in some scenarios in which there is a predisposition to respond. As anti–PD-1 has already received FDA approval and has been used more extensively in glioblastoma (49–51), a clinical trial of this combination may be warranted in a subset of glioblastoma patients. Since 8803 would be a first-in-human study, a phase I clinical trial with intratumoral administration of 8803 into recurrent WT glioblastoma patients would first need to be conducted to define an MTD/MED. We also intend to conduct a window-of-opportunity analysis to enable immune profiling of posttreatment glioblastoma tissue and to ascertain whether STING promoter methylation status correlates with radiographic responses to 8803.

The work described here has direct clinical translational relevance for treating patients with glioblastoma. STING agonists have the distinct elements necessary to modulate the TME to eradicate the tumor that prior immunotherapeutic monotherapies have been unable to achieve adequately. This, on a background of preclinical safety and tolerability, lends strong support for further investigation of this approach in the clinic. As this approach advances, careful monitoring of patients for acute inflammatory toxicities, including marked cerebral edema, encephalitis, and cytokine release syndrome, will be essential. While the preclinical models provide reassurance with their lack of demyelination and neurodegeneration, assessing these subacute and chronic toxicities will be essential. This will be of particular importance if the long-term survival/cure findings observed in the preclinical models can be recapitulated in even a portion of the patient population.

It is relevant to mention that in the clinical scenario in which chromosomal instability (CIN) is a driver of cancer metastasis, chronic activation of the cGAS/STING pathway can ultimately lead to IFN tachyphylaxis, and the reversal of CIN or depletion of cancer cell STING can reduce metastasis of melanoma, breast, and colorectal cancers (52). Our studies indicate that in cancers such as glioblastoma in which the STING pathway is deactivated within the tumor cell and thereby lacking chronic activation, a STING agonist provides a beneficial therapeutic response. However, repeated and prolonged dosing of STING agonists may ultimately result in a diminished response. Therefore, clinically, a limited number of doses would be given to patients necessary to acutely induce an antitumor response, thereby avoiding a chronic activation scenario. Notably, in the QPP8 model in which STING is activated, the application of a STING agonist is curative, suggesting different cancer cell lineage responses to STING modulation and the need for a companion biomarker.

The safety and tolerability observed with this approach in non-CNS malignancies provides reassurance (53, 54). When designing clinical trials using a STING agonist–based approach, there will be value in assessing the “tail of the curve” for longer-term survivors, in addition to evaluating other key endpoints such as median overall survival. If efficacy is demonstrated, preclinical and clinical correlative studies may help elucidate predictive biomarkers, allowing clinicians to tailor their therapeutic approach for this patient population.

## Methods

### Sex as a biological variable.

Our study examined male and female animals, and similar findings are reported for both sexes.

### Animal models.

Humanized NOD.Cg-*Prkdc^scid^ Il2rg^tm1Sug^* Tg(SV40/HTLV-IL3,CSF2)10-7Jic/JicTac (huNOG-EXL, model HSCCB-13395-F) were purchased from Taconic Biosciences. To induce intracerebral tumors in huNOG-EXL mice, indwelling specialty cannulas (Protech International) were implanted. U87 cells were collected in the logarithmic growth phase using trypsin EDTA, washed, resuspended with PBS, and loaded into a 25 μL Neuros syringe (65460-10, Hamilton) with an attached 33-gauge needle. The cannula implantation site was positioned 2 mm behind and to the right of the bregma and 4 mm below the surface of the skull at the coronal suture. The tumor cells were implanted through the cannula using a stereotactic frame (Kopf Instruments). The i.c. tumorigenic dose was 1.5 × 10^5^ for U87 cells in a total volume of 2.5 μL.

A similar strategy was used for the immunocompetent models. WT C57BL/6J (strain 000664) were purchased from The Jackson Laboratory. WT C57BL/6J mice were implanted with a cranial cannula (Protech International) that is compatible with a 26-gauge 1700 series 10 μL gas-tight Hamilton syringe (80075, Hamilton) loaded onto a multi-injector system with an infusion rate of 0.5 μL/min (PHD 2000 syringe pump, 70-2000, Harvard Apparatus). To induce intracerebral tumors in C57BL/6J mice, GL261, CT-2A, QPP4, and QPP8 (including 2 subclones, QPP8v and QPP8v2) cells were collected in the logarithmic growth phase using trypsin EDTA (GL261 and CT-2A) or Accutase (Corning) (QPP4, QPP8), washed, and resuspended in PBS. The intracerebral tumorigenic dose was 5 × 10^4^ for GL161, 1 × 10^5^ for CT-2A, 2.0 × 10^5^ for QPP4, 3.0 × 10^5^ for QPP8, and 2.0 × 10^5^ for QPP8v and QPP8v2 subclones in a total volume of 2.5 μL (GL261 and CT-2A) or 5 μL (QPP4 and QPP8).

For RAG^–/–^ mice (B6.129S7-*Rag1^tm1Mom^*/J, stock 002216, The Jackson Laboratory) implanted with QPP8 and QPP8v tumors, tumor outgrowth through the cannula prevented the use of a cannula system. Mice were instead implanted and treated using a stereotactic device through a burr hole drilled 2 mm right lateral and 2 mm anterior to the bregma at a depth of 4 mm. The burr hole was sealed with bone wax following tumor implantation and i.c. treatments to prevent extracranial tumor outgrowth.

Mice were then randomly assigned to vehicle control or treatment groups. The mice were observed daily for survival recording and were compassionately euthanized upon signs of neurological deficit (lethargy, hypothermia, failure to ambulate, lack of feeding, body condition score <2.0, or loss of >20% body weight).

To generate the aggressive QPP8 subclone, 10 mice were stereotactically implanted with QPP8, and 6 tumors were harvested as the mice developed neurological symptoms. These tumors were placed into a cell suspension, washed, and cultured in appropriate media. Stable neurospheres were generated in vitro for 3 of 6 tumors, and then tested for in vivo penetrance and kinetics (Supplemental Figure 2). On day 215, all remaining mice were euthanized and there was no histological evidence of gliomas.

### In vivo treatments.

The STING agonist 8803 was administered i.c. via cannula usually once a week (see figures or legends for treatment schemas) for 2 weeks at a concentration of either 2.5 or 5 μg/mouse starting on day 7 or 10 (U87, GL261, CT-2A, and QPP8v) or on day 14 (QPP4, QPP8, and QPP8v2) after tumor implantation. The anti-NK1.1 antibody (NK cell–depleting antibody, BioXCell, clone PK136) was administered at 250 μg/mouse, 2 doses per week for 7 weeks, with the first dose at day 5 after tumor cell implantation, 48 hours before the STING agonist treatment. Anti–PD-1 antibody (BioXCell, clone RMP1-14) was administered at a concentration of 200 μg/mouse i.p., 3 times per week (M/W/F), for 2 weeks (CT-2A), or at a concentration of 250 μg/mouse i.p. and 25 μg/mouse i.c. on days 7, 10, and 13 (QPP8v). WP1066 (20), which blocks p-STAT3 (55), was supplied in-house and from Medchem Express (HY-15312). WP1066 does not influence JAK2 kinase activity at concentrations up to 10 μmol/L based on KINOME scan profiling (22). The IC_50_ of WP1066 for GL261 is 4.91 μmol/L. For in vivo treatment, the mice were treated via oral gavage with WP1066 (30 or 60 mg/kg) in a vehicle of DMSO/PEG 300 (20 parts/80 parts) or vehicle control on a M/W/F schedule for either 1 or 3 weeks, starting on day 7 after tumor cell implantation.

### Online data sets analysis.

A cohort of 142 patients with IDH-1–WT glioblastoma from TCGA data set was obtained from GlioVis (http://gliovis.bioinfo.cnio.es/ Accessed March, 2023.). Kaplan-Meier curves using log_2_-transformed mRNA expression of selected markers (STING [*TMEM173*], *IRF3*, *TBK1*, *STAT3*, and PD-1 [*PDCD1*]) were downloaded and analyzed using GlioVis’s built-in analysis tools. Additionally, pediatric glioma data from the Greisinger database (Accessed March, 2023.) was extracted for log_2_-transformed mRNA expression of STING between glioblastoma and normal brain (27). Spatial RNA-seq data were collected from the Ivy Glioblastoma Atlas Project (IVY-GAP; https://glioblastoma.alleninstitute.org/ Accessed March, 2023.), which contains 41 patients whose tumor samples were classified based on anatomic features. *Z*-score normalization of RNA-seq expression of STING, IRF3, TBK1, STAT3, and PD-1 was downloaded and visualized using scatter plots. A publicly available scRNA-seq database from Abdelfattah et al. (56) was analyzed using the online platform through the Broad Institute to create dot plots of key immune markers. This database contains the following cell types (and counts): a-Mic (2,594 cells), AP-Mic (3,303 cells), CD4^+^ T cells (1,829 cells), CD8^+^ T cells (9,132 cells), DCs (1,715 cells), h-Mic (14,851 cells), i-Mic (14,851 cells), MDSCs (7,206 cells), CD45^–^ cells (14,852 cells), naive T cells (3,803 cells), NK cells (1,068 cells), proliferating macrophages (1,152 cells), suppressive macrophages (s-mac) 1 (114,851 cells), s-mac 2 (26,142 cells), and Tregs (2,651 cells). The entire scRNA database can be interactively explored at https://singlecell.broadinstitute.org/single_cell/study/SCP1985/

### Statistics.

All statistical analyses were performed using GraphPad Prism software version 9.4.0. The Mann-Whitney *U* test was used to assess the significance of differences between 2 groups. All the data are reported as mean ± SEM. Multiple groups were analyzed with 1-way ANOVA along with Tukey’s multiple-comparison post hoc test and compared to control. Survival analysis between control and experimental groups was determined by the Kaplan-Meier method, and statistical significance was assessed using the log-rank test (Mantel-Cox). The *P* values for curve comparisons were calculated using the log-rank method followed by Bonferroni’s correction. A *P* value of less than 0.05 was considered significant: *P < 0.05; **P < 0.01; ***P < 0.001; ****P < 0.0001; NS, not significant.

### Study approval.

All in vivo mouse experiments were approved in accordance with Laboratory Animal Resources Commission Standards by the Institutional Animal Care and Use Committee (IACUC) and conducted according to the approved protocols 08-06-11831 and 0001378-RN01/RN02 at MD Anderson Cancer Center, and IS00020425 at Northwestern University. Human glioblastoma specimens were collected from consented patients who received surgery at Northwestern Memorial Hospital according to the approved Institutional Review Board protocol STU00214485.

### Data availability.

The data used to support the findings of this study are available within this article and within the Supporting Data Values file.

## Author contributions

HN, STL, CHC, MAC, and ABH designed experiments. HN, STL, ST, LH, CHC, IW, MS, MB, GH, CD, SP, CD, CLC, KM, AS, J Walshon, MO, J Wei, and AM developed methodology and carried out experiments. JH developed methodology. CMH performed histological examination of specimens. WP1066 was partly supplied by WP. DMA, LCP, RS, CDJ, MSL, MAC, and ABH acquired funding. HN, STL, MB, IB, RVL, CMH, DMA, MAC, and ABH analyzed data. HN and ABH wrote the initial draft of the manuscript, which was reviewed by all authors. The final manuscript was approved by all authors. The co–first authors, HN and STL, equally contributed to data generation and analysis; HN and ABH were responsible for generating the first drafts of images and text.

## Supplementary Material

Supplemental data

Unedited blot and gel images

Supplemental video 1

Supporting data values

## Version 1 06/17/2024

Electronic publication
