# Supplementary material for: STING agonist 8803 reprograms the immune microenvironment and increases survival in preclinical models of glioblastoma
Source: J Clin Invest. 2024 Jun 17;134(12):e175033. doi: 10.1172/JCI175033 (PMC11178548; doi:10.1172/JCI175033)
Supplement: Supplemental data [file jci-134-175033-s161.pdf]

Supplementary Methods

Cell lines

GL261, CT-2A and U87 cell lines were maintained in Dulbecco's Modified Eagle Media (DMEM; Corning) with 10% fetal bovine serum (FBS). Human Umbilical Vein Endothelial Cells (HUVEC) (Clonetics) were maintained in Endothelial Cell Growth Medium 2 media supplemented with EGF (5ng/ml), FGF (10ng/ml), IGF (20ng/ml), VEGF 0.5ng/ml, Ascorbic Acid (1µg/ml), Heparin (22.5µg/ml), and Hydrocortisone (0.2 µg/ml) (Sigma). The THP1 cell line (ATCC) was cultured in RPMI 1640 (Corning). QPP4 and QPP8 (including subclone) cell lines were maintained in DMEM/F12 media with B-27 supplement (Gibco), supplemented by EGF (20ng/ml), and FGF (20ng/ml) (StemCell Technologies). QPP4 and QPP8 are glioblastoma cell lines derived from spontaneously arising Qk/Trp53/PTEN triple-knock out mice [25] that recapitulate the immune suppressive myeloid-dominant nature of the tumor microenvironment of glioblastoma [26]. The U87 cell line was sourced from ATCC [27-29]. All cell lines were determined to be mycoplasma negative prior to implantation. U87, CT-2A, and GL261 *in vitro* all express low levels of total STING compared to other human and mouse cell lines on Western blot analysis. U87 has high methylation at a single CpG site (~80-90%), whereas CT-2A and GL261 are not methylated at the homologous mouse CpG site. In murine cell line testing, there is insufficient evidence that STING is controlled at the methylation level in the mouse.

Analysis of murine tumor immune infiltrates

QPP8 and U87 tumors were implanted and treated. Following euthanasia, tumors, cervical lymph nodes, and naïve spleen control were harvested, diced, and placed into 70-micron filters in 6 cm plates containing tumor digestion media (serum-free RPMI containing collagenase H (1 mg/mL, Sigma) and DNAse (160 µg/mL, Roche) for 30 min at 37℃. Tumors were mashed through filters, and single-cell suspensions were washed with complete RPMI. Myelin was removed via Percoll density gradient centrifugation (Sigma-Aldrich). Samples were transferred to 96-well round bottom plates for flow cytometry staining, then stained with Near IR live/dead fixable viability dye (Invitrogen), washed, and fixed overnight using the eBioscience fixation/permeabilization kit (ThermoFisher). Samples were stained with fluorescently labeled antibodies for flow cytometry analysis.

Flow Cytometry

For intracellular staining, samples were washed in U- bottom 96-well plates with FACS buffer, then stained 10 min at 4℃ with Near IR live/dead fixable viability dye (Invitrogen). Samples were washed with FACS buffer, then fixed/permeabilized with eBioscience fixation/permeabilization kit (ThermoFisher). Cells were washed in permeabilization buffer (ThermoFisher) and incubated 15 min at 4℃ with anti-mouse CD32/CD16 Fc- block antibody (Leinco), followed by incubation for 1 h at 4℃ with fluorescently labelled antibodies (**Supplementary Table 1, 2,** and **3**). Following staining, samples were washed and analyzed using a BD LSRFortessa X-30 prototype and the Cytek Aurora flow cytometers. Flow cytometry data were analyzed using the FlowJo v7.6.5 and v10.7 application (BD) and Cytobank premium (Beckman Coulter life sciences). Negative values in multiparameter flow cytometry data analysis can occur in data acquisition due to adjustment of the original measurements by the instrument hardware and software [32]. Gating strategies are shown in **Supplementary Figure 6** and **7**.

In vitro immune cell polarization

Immune cells were treated for 48h with IL-4 (10 ng/mL), IL-13 (20 ng/mL), and TGF-β (20 ng/mL) to induce skewing to M2, then harvested, washed, and treated for 48h with the STING agonist 8803 (10 ug/mL). The cell phenotype was then characterized using flow cytometry at 24, 48, and 72 h after STING treatment. For the bone marrow-derived myeloid cells (BMDMs), polarization was achieved using IL-4 (20 ng/ml) monotherapy for 48h.

Antigen-specific OT-1 cell proliferation

Mouse T cells were isolated from the spleens of WT or OT-1 C57BL/6 mice using the Mouse T cell isolation kit (StemCell Technologies, Cat no. 19853) [31]. The WT and OT-1 T cells were labeled with eBioscience Cell Proliferation Dye eFluor 450 (5 μM concentration) (Thermo Fisher) and then incubated with IL-2 (50 U/mL; Peprotech) with or without 1, 5, or 10 μM of STING 8803 for 24 h. The co-culture was set up in a 96-well plate at a 5:1 ratio (T cell:GL261 OVA) and maintained for 72 h in the CO_2 incubator. After 72 h, the T cells were collected, blocked with CD16/32, and stained with the CD8a BV605 antibody (Biolegend Clone 53-6.7, 1:200 dilution) and Fixable viability dye eFluor780. The stained cells were acquired by the BD Symphony and analyzed by FlowJo™ v10.7.1 software.

Tumor cell Matrigel invasion assay

For this assay, the Matrigel-coated inserts with 12-well companion plate (Corning® BioCoat™ Matrigel® Invasion Chamber, Cat# 354480) were used. The GL261 cells were plated and treated

with the STING agonist 8803 (1, 5, and 10 μM) for 24 h. Cells were harvested, washed with DMEM medium (Corning), seeded on the upper chamber of the inserts (8 μm pore size), and incubated for 22 h. 10,000 cells/condition in 0.5 mL of DMEM were added to the upper chamber and 0.5 ml of 20% FBS-containing DMEM was added to the lower chamber. After 22 h, the upper chambers were swabbed to completely remove the cells that had not migrated and then fixed in 4% PFA overnight. The next day, the PFA was discarded, and the lower side of the insert was stained with 0.1% Neutral red for 20 min at room temperature. The excess stain was removed by washing several times with distilled water and then the insert was separated from the chamber. The insert was mounted on glass slides, sealed using nail polish, and imaged using a brightfield microscope for subsequent analysis.

Western blotting analysis

HUVEC, GL261, CT-2A, U87, QPP4, QPP8, and THP1 cell lines were lysed in RIPA buffer (Thermo Scientific) supplemented with Halt™ Protease and Phosphatase Inhibitor Cocktail (Thermo Scientific) and Benzonase (MilliporeSigma). Proteins (20ug) were separated with a 4-12% Bis-Tris gel (Invitrogen) in 1 X MOPS Buffer (Invitrogen) gel electrophoresis, transferred to a polyvinylidene difluoride membrane (Bio-Rad), and probed with antibodies against STING (Cell Signaling; #13647) and β-Actin (Cell Signaling; # 5125). THP1 cells were treated with 8803 and WP1066 for 8 and 4 h, respectively.

Ubiquitination pull-down assay

Ubiquitination of STING was measured using the Signal-Seeker Ubiquitination Detection Kit (BK161, Cytoskeleton) according to the manufacturer's protocol. Briefly, THP-1 cells were treated with 8803 and WP1066 for 8 and 4 h, respectively, and incubated with 10 μM of proteasome inhibitor (MG132) during the last 6 h, then washed with cold PBS and lyzed with BlastR lysis buffer supplemented with protease, de-ubiquitination, and SUMOylation inhibitors. The lysates were passed through the BlastR filter and diluted with BlastR dilution buffer, followed by incubation with ubiquitination affinity beads for 6 h at 4°C. The beads were washed with BlastR-2 buffer and eluted by elution buffer. Ubiquitination of STING was evaluated by western blot analysis.

Tissue acquisition and preparation

Under the approved IRB protocol STU00214485, newly diagnosed glioblastoma IDH WT patient tumor samples were collected from the operating room at Northwestern Memorial Hospital and formalin-fixed and paraffin-embedded (FFPE). FFPE slides at a thickness of 4 μm were prepared

by the Neurological Surgery Tumor Bank at Northwestern University on positively charged slides and stored at room temperature for the subsequent analysis. After compassionate euthanasia according to the standard guidelines of the IACUC, mice brains were collected and fixed in formalin for FFPE slide preparation by the Pathology Core, Northwestern University. For each case, H & E slides were reviewed, and the tissue was segmented by a certified neuropathologist (CMH). FFPE slides were pre-processed for antigen retrieval using the PT Module (Epredia) with Dewax and HIER Buffer H (TA999-DHBH, Epredia) for 60 min at 102°C. Subsequently, slides were rinsed and stored in a Multi-staining Buffer (BU06, Lunaphore Technologies) till use.

Immunohistochemistry (IHC) and Luxol fast blue staining

Luxol Fast Blue-Cresyl Echt Violet stain was performed according to standard protocol [33]. Slides were also processed for chromogenic IHC for CD3ε (rabbit monoclonal IgG; Abcam, Cat# ab16669; dilution: 1:250). An anti-rabbit antibody-horseradish peroxidase (HRP) polymer conjugate (MACH2, Biocare) was used in conjunction with the chromogenic substrate 3,3'-diaminobenzidine to visualize the primary antibody binding sites. IHC slides were counterstained with hematoxylin, mounted, and stored at room temperature until analyzed.

Multiplexed SeqIF™ staining

All antibodies were validated using conventional immunohistochemistry and/or immunofluorescence (IF) staining, in conjunction with the corresponding fluorophores and the spectral 4',6-diamidino-2-pheynlindole (DAPI) counterstain. All antibodies were tested at three different dilutions for optimal concentration and best signal/noise ratio, starting with the manufacturer-recommended dilution (MRD), then MRD/2, and MRD/4. Secondary Alexa fluorophore 555 (ThermoFisher Scientific) and 647 (ThermoFisher Scientific) were used at 1/200 and 1/400 dilutions respectively. The antibodies used in the human and mouse multiplex panels are listed in **Supplementary Table 4**. The optimizations and full runs of the multiplex panel were executed using the Sequential Immunofluorescence (SeqIF™) methodology integrated in the Lunaphore COMET™ platform (characterization 2 and 3 protocols, and SeqIF™ protocols, respectively). The SeqIF™ protocol is based on automated iterative cycles of antibody-based staining, followed by imaging, and elution of the primary and secondary antibodies. The quenching step was performed using Quenching buffer (BU08-L, Lunaphore Technologies®) for 30 s at 37˚C. Antibody staining time was 4 mins for primary antibodies and 2 mins for the secondary antibodies at 37˚C. The imaging step was performed using Imaging buffer (BU09, Lunaphore Technologies®) with specific exposure times for DAPI, TRITC, and Cy5 channels

using an integrated fluorescent high-power field microscope (20x magnification). The elution step was performed using Elution buffer (BU07-L, Lunaphore Technologies®) for 2 mins at 37˚C. The staining can be performed at a maximum of 4 tissue slides simultaneously where no sample manipulation is required. Image registration was executed immediately after concluding the staining procedure by COMET™ control software. The integrated COMET™ microscope captures the fluorescent signals (DAPI, TRITC, and Cy5) separately at the corresponding fluorophore wavelength, with preset exposure times, and then these captures were registered in a single OME.TIFF file without disrupting the unique fluorescent spectral signature of the markers. The OME.TIFF images were analyzed and exported in Lunaphore Viewer. The quantification analysis was then conducted using the guided workflow and Phenoplex feature on the Visiopharm® platform.

Supplementary Table 1 – Conjugated antibodies used for flow cytometry analysis from the QPP model.

TARGET	FLUOROPHORE	CLONE
**CD8A**	BUV395	53-6.7
**CD80**	BUV496	16-10A1
**LY6G**	BUV563	1A8
**KI67**	BUV615	B56
**CD45**	BUV661	30F11
**TCRB**	BUV737	H57-597
**CD4**	BUV805	GK1.5
**CD163**	eF450	TNKUPJ
**F4/80**	BV480	T45-2342
**NK1.1**	BV570	PK136
**CD11B**	BV605	M1/70
**CD223 (LAG3)**	BV650	C9B7W
**PD-L1**	BV711	MIH5
**CD11C**	BV750p	N418
**PD-1**	BV786	RMP1-30
**INOS**	Alexa488	CXNFT
**SIRPA**	BB700	P84
**IA/IE**	BB790	M5/114.15.2
**ARGINASE**	PE	A1exF5
**CD206**	Alexa594	C068C2
**CD86**	PE-Cy5	GL-1
**GRANZYME B**	PE-Cy5.5	NGZB
**TMEM119**	PE-Cy7	V3RT1G0sz
**FOXP3**	APC	FJK-16S
**LY6C**	AF700	AL-21
**VIABILITY DYE**	Near-IR	Invitrogen Live/Dead fixable near-IR viability dye

Supplementary Table 2 - Conjugated antibodies used for flow cytometry analysis from the U87 model.

ANTIGEN	FLUOROPHORE	CLONE
**HCD11C**	BUV395	B-Ly6
**HCD8**	BUV496	RPA-T8
**LY6G**	BUV563	1A8
**KI67**	BUV615-P	B56
**CD45**	BUV661	30-F11
**CD3**	BUV737	UCHT1
**HCD45**	BUV805	HI30
**CD15**	BV421	HI98
**F4/80**	BV480	T45-2342
**HLA-DR**	BV570	L243
**CD14**	BV605	M5E2
**HCD4**	BV650	SK3
**CD68**	BV711	Y1/82A
**MHC IA/IE**	BV750-P	M5/114.15.2
**HPD-L1**	BV786	MIH1
**HCD206**	BB515	19.2
**CD11B**	BB660-P	M1/70
**HCD19**	BB700	SJ25C1
**HCD163**	BB790-P	GHI/61
**CX3CR1**	PE	SA011F11
**ARGINASE**	Alexa 594	8C9
**CD33**	PE-CY5	WM53
**CD11C**	PE-CY7	HL3
**TMEM119**	Alexa 647	106-6
**LY-6C**	Alexa 700	AL-21
**HCD80**	APC-H7	L307.4

Supplementary Table 3 - Conjugated antibodies used for flow cytometry analysis of the BMDM cells.

MARKER	FLUOROPHORE	CLONE
**CD101**	NovaFluor Yellow 730	Moushi101
**CD11B**	PE-Dazzle 594	M1/70
**CD11C**	Spark Blue 550	N418
**CD197/CCR7**	PE/Cyanine5	4B12
**CD204/MSR**	Alexa Fluor 488	M204PA
**CD206/MMR**	Brilliant Violet 421	C068C2
**CD45**	Brilliant Violet 750	30-F11
**LAMP1/CD107A**	APC-Fire 750	1D4B
**LY-6C**	Brilliant Violet 510	HK1.4
**LY-6G**	Brilliant Violet 605	1A8
**MHC II (IA/IE)**	Alexa Fluor 647	M5/114.15.2
**PD-1/CD279**	Brilliant Violet 480	J43
**TIM3**	Brilliant Violet 711	B8.2C12
**ARGINASE 1**	eFluor 450	A1exF5
**GRANZYME B**	PE-Cy7	QA16A02
**IFN-γ**	APC	XMG1.2
**TNF-α**	Brilliant Violet 785	MP6-XT22
**LIVE/DEAD YELLOW**	ThermoFisher L34959	

Supplementary Table 4 - Unconjugated antibodies used for multiplex immunofluorescence staining on the COMET system (Lunaphore technologies).

ANTIGEN	MANUFACTURER	CAT#	CLONE
**GFAP**	Abcam	Ab68428	EPR1034Y
	Sigma	MAB360	GA5
**CD31**	Abcam	Ab225883	EPR17259
**CD4**	Abcam	AB133616	EPR6855
	Abcam	Ab183685	EPR19514
**CD8**	Leica	PA0183	4B11
	Cell signaling	98941	D4W2Z
**CD68**	Dako Agilent	GA613	PG-M1
	Santa Cruz	Sc-20060	KP1
**CD163**	Abcam	Ab182422	EPR19518
**CD206**	Abcam	Ab64693	Polyclonal
**CD11C**	Abcam	Ab52632	EP1347Y
	Cell signaling	97585	D1V9Y
**P2RY12**	Atlas antibodies	HPA014518	Polyclonal
	Cell signaling	69766	E9J1J
**HLA-DR**	Abcam	Ab20181	TAL 1B5
**MHC II**	Abcam	Ab23990	MRC OX-6
**STING**	Cell signaling	13647S	D2P2F
**p-IRF3**	Cell signaling	4947S	4D4G
**p-TBK1**	Cell signaling	5483S	D52C2
**p-STAT3**	Cell signaling	9145S	D3A7 (Tyr705) XP
**PD-1**	Abcam	Ab137132	EPR4877(2)
	Abcam	Ab214421	EPR20665

Supplementary Figures

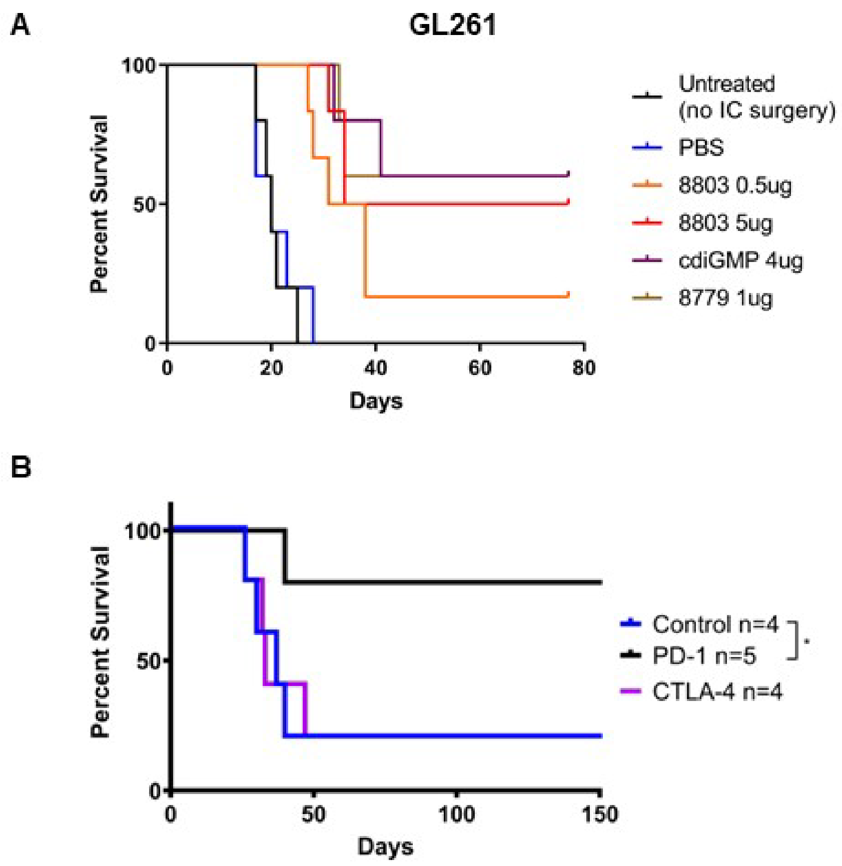

Supplementary Figure 1: Signal finding of 8803 in the GL261 model. A, Kaplan–Meier method showing survival outcomes in C57BL6 mice implanted with GL261 (n=5-6 mice/group) and then treated with a single dose of PBS, 8803, 8779, or cdiGMP at the concentration designated in the legend on day 10. Cyclic di-GMP (cdiGMP) serves as the positive control but is not scalable for use in clinical trials. **B**, Kaplan-Meier method showing the survival outcomes of C57BL6 mice implanted with GL261 and then treated with either anti-PD-1 (n=5), anti-CTLA-4 (n=4), or were left untreated. Unlike human glioblastoma patients, the GL261 preclinical model is responsive to anti-PD-1. Occasional survivors in the GL261 model in control groups can occasionally be observed and are reflective of the baseline immunogenicity.

A

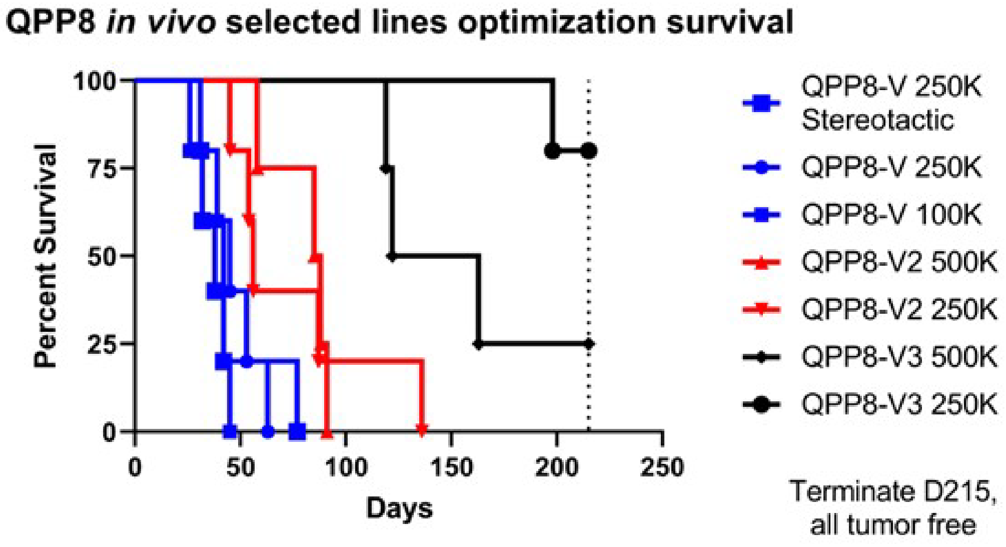

B

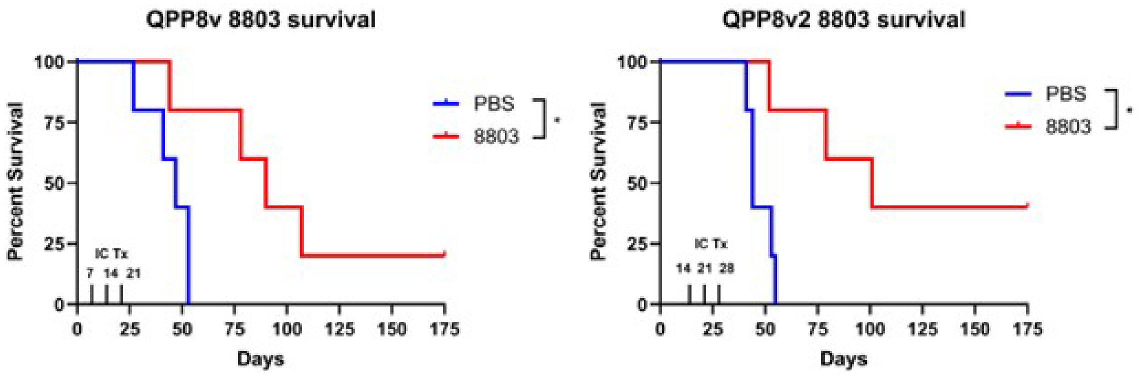

Supplementary Figure 2: QPP8 subclones and therapeutic activity of 8803. A, Three subclones were created and evaluated for *in vivo* growth kinetics as a function of cell dose using the Kaplan–Meier method**. B**, The survival rate of C57BL/6 mice with intracerebral QPP8v subclone treated with 8803 (5 ug) on days 7, 14, and 21 (left panel). The survival rate of C57BL/6 mice with intracerebral QPP8v2 subclone treated with 8803 (5 ug) on days 14, 21, and 28 (right panel). Because the median survival time was predicted to be longer for the v2 clone, the treatment schedule was initiated one week later relative to the v subclone.

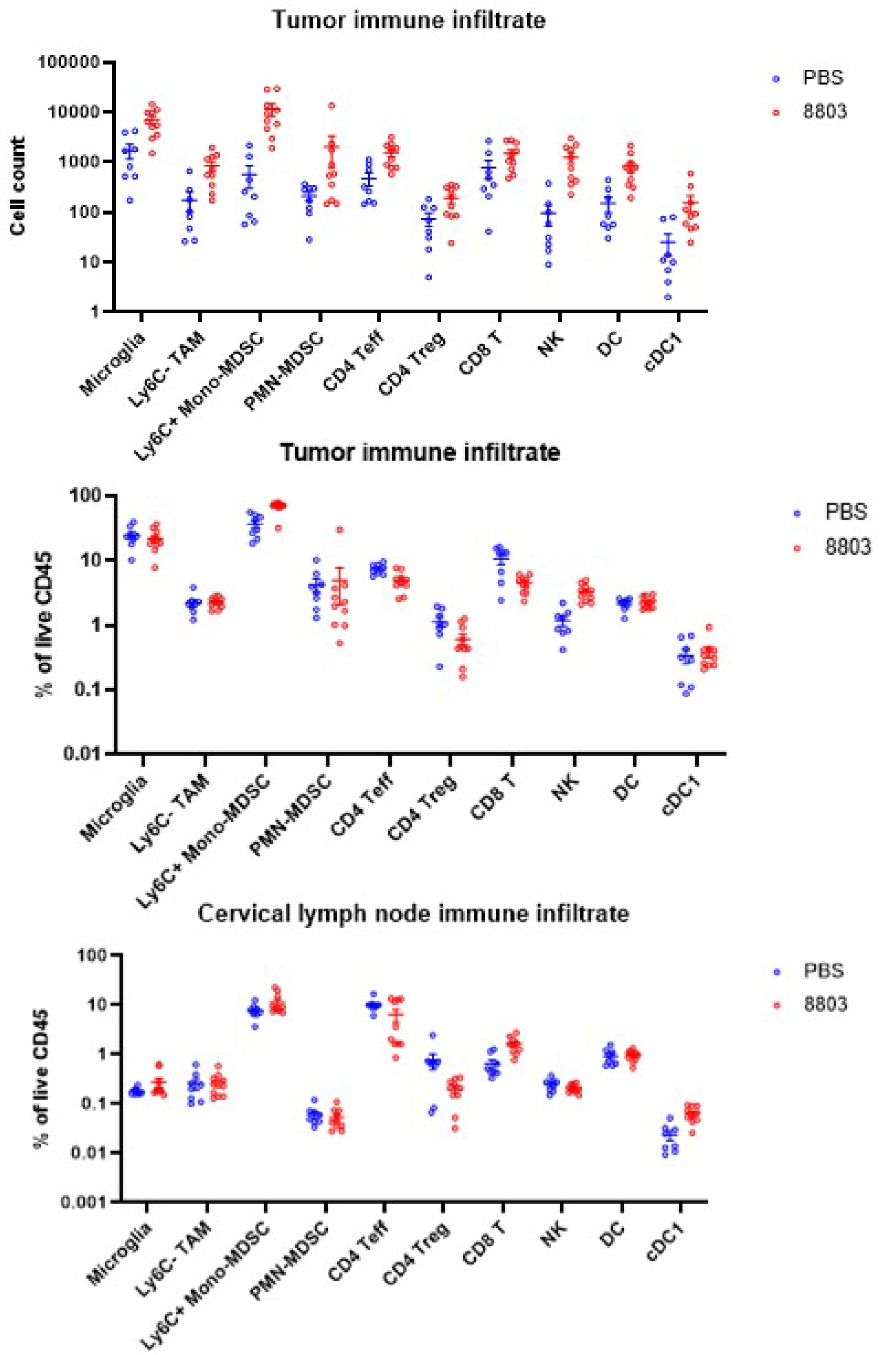

Supplementary Figure 3: Flow cytometry analysis from QPP8-infiltrating and cervical lymph node immune cells using the BD LSRFortessa X-30 prototype flow cytometer. Cell lineages identification was based on markers from Supplementary Table 1 and gating strategy in Supplementary Figure 2, then quantified as a function of treatment with either PBS or 8803. Tumors were treated on days 60 and 67, and then specimens were analyzed 48 hours after the last dose.

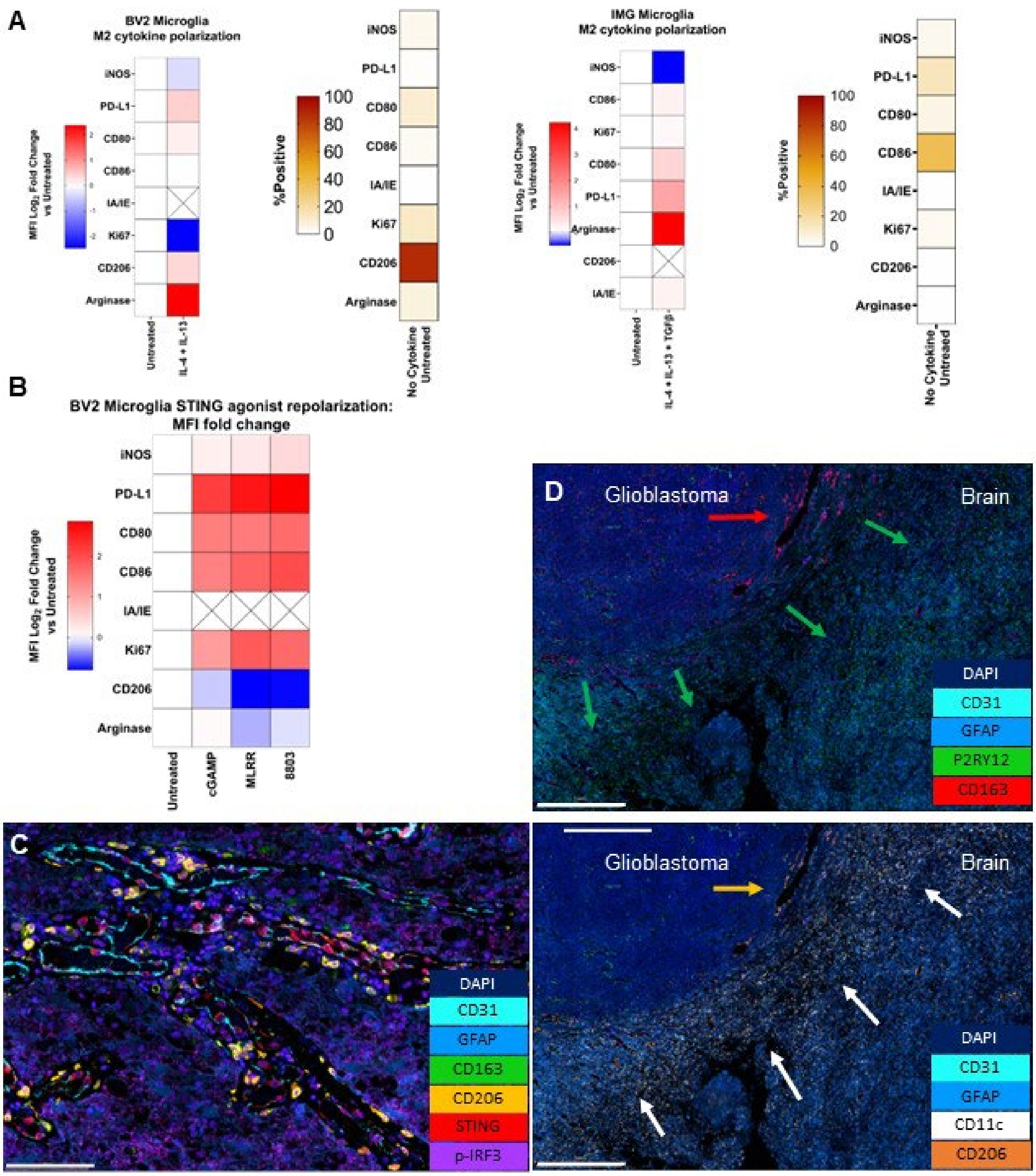

Supplementary Figure 4: Microglia profiles and glioblastoma TME. **A**, Baseline and IL-4 and IL-13 polarized BV2 microglia or IL-4, IL-13, and TGF-β polarized IMG microglia phenotype. **B**, BV2 microglia were treated with STING agonists with increasing potency from cGAMP, MLRR-S2-CDA, to 8803 and profiled based on various markers. These were quantified based on MFI fold change or percentage of cells that are positive and then presented as a heat map. **C** and **D**, Representative multiplexed sequential immunofluorescence imaging of human glioblastoma

showing: **C**, perivascular localization of CD163+CD206+ macrophages expressing cytoplasmic STING and nuclear p-IRF3 at the edge of the tumor. Scale bar 100 μm; **D**, Low magnification view of the glioblastoma microenvironment, from tumor enriched with CD163+ macrophages (top image, red arrow), to edge with CD206+ perivascular macrophages and CD11c+ antigen presenting cells (bottom image, orange, and white arrows, respectively), to peritumoral brain with P2RY12+ microglia (top image, green arrows). Scale bars 1 mm.

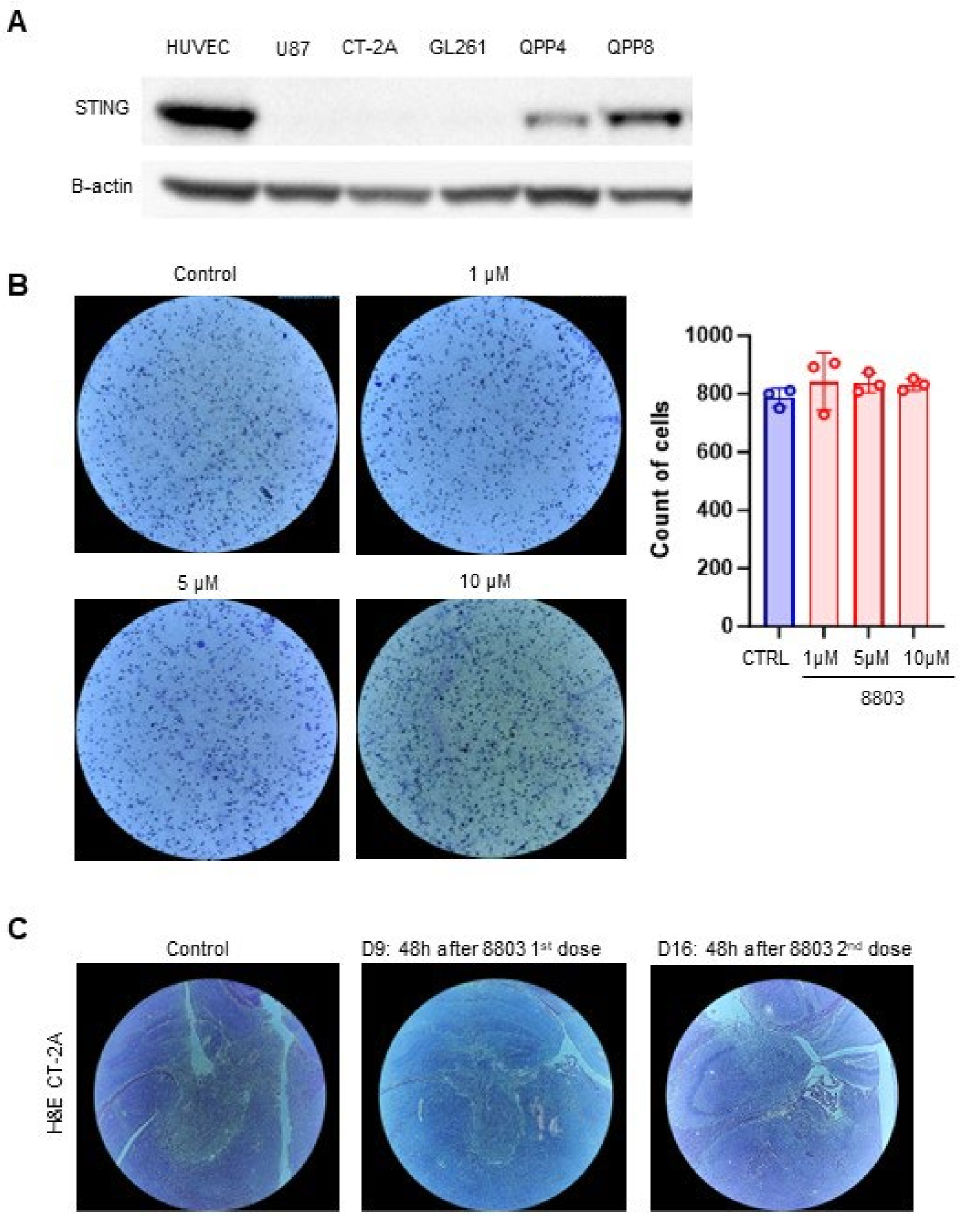

Supplementary Figure 5: Characterization of STING expression in gliomas cells and invasion upon treatment with 8803. A, Western blot analysis of STING expression in cell lines HUVEC, U87, CT2A, GL261, QPP4, and QPP8. Actin control is shown for protein loading. **B**, Matrigel invasion assay of GL261 glioma cells treated with STING agonist 8803 (1, 5, and 10 μM) showing images of the invaded chambers stained with neutral red (panels on the left, 10x magnification) and the number of invaded cells represented in the column dot plot. **C**, Hematoxylin and eosin images of the CT-2A glioma cells implanted intracranially in C57BL6 mice treated with STING agonist 8803. 4x magnification.

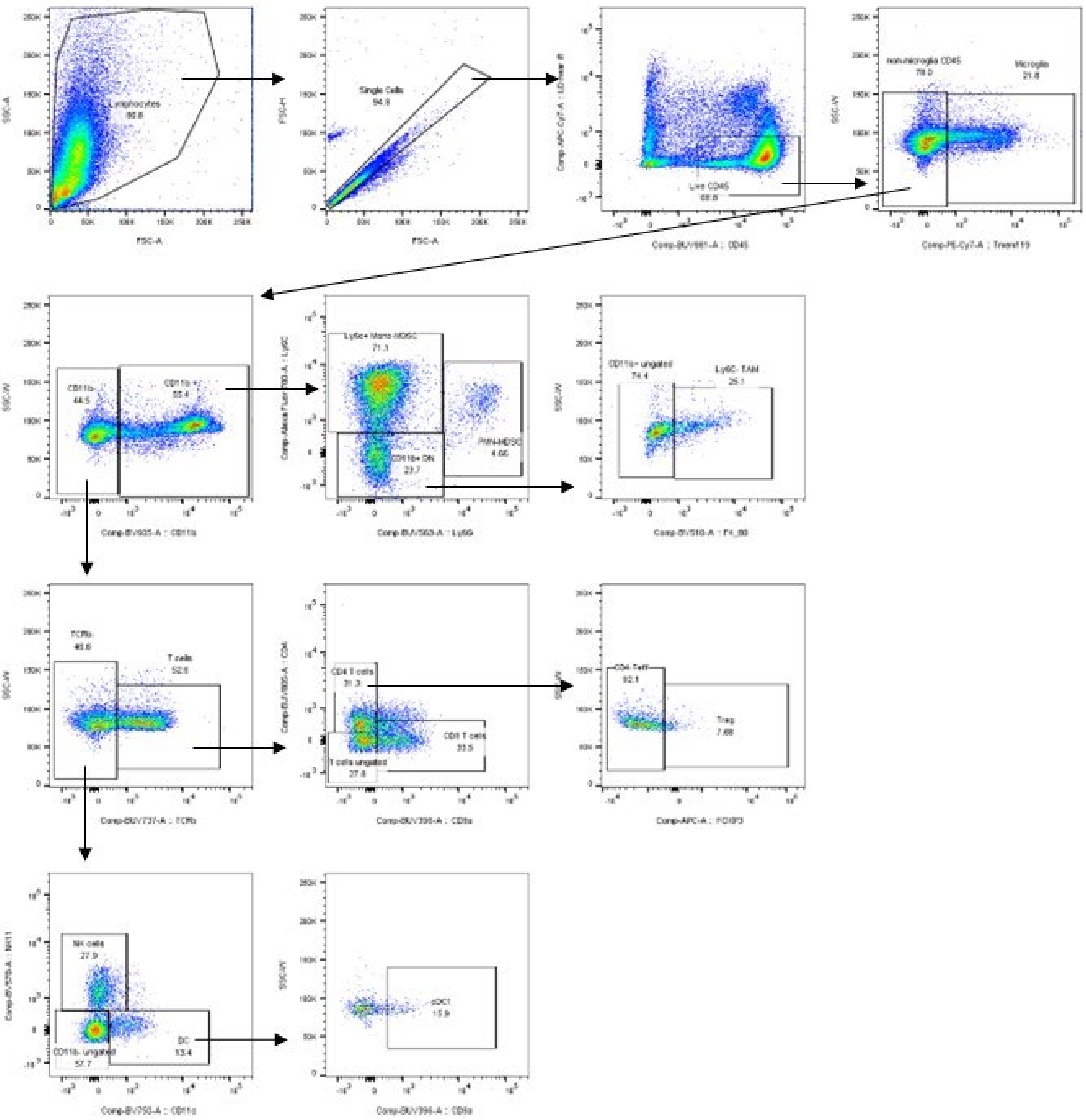

Supplementary Figure 6: Representative example of the gating strategy of the flow cytometry analysis conducted on tumor-infiltrating immune cells from murine brains implanted with QPP8. Debris and doubles/aggregates were removed and then live CD45+ immune cells were identified. CD45+ cells were divided into Tmem119+ microglia and Tmem119- non-microglia. Non-microglia were divided into CD11b+ and CD11b-. CD11b+ cells were divided into Mono-MDSC defined as Ly6C+/Ly6G-, PMN-MDSC defined as Ly6G+, and TAM defined as Ly6C-/Ly6G-/F4-80+. CD11b- cells were further divided into TCRb+ T cells and TCRb- cells. T cells were divided into CD4-/CD8+ T cells, CD4+/CD8-/FOXP3- Teffs, and CD4+/CD8-/FOXP3+

Tregs. TCRb- cells were divided into NK1.1+ NK cells, and CD11c+ Dendritic Cells. cDC1 cells were further defined as CD11c+/CD8a+.

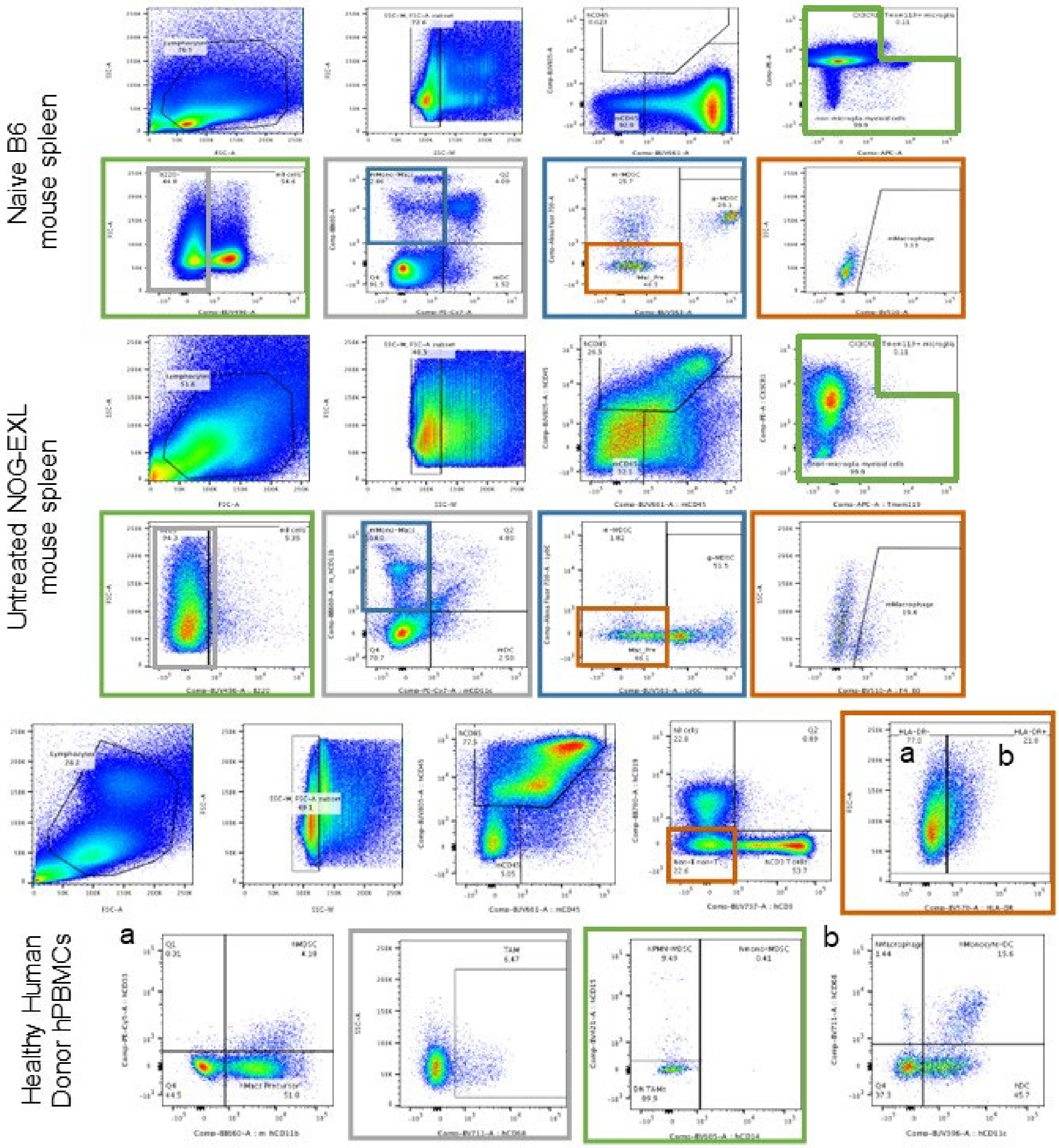

Supplementary Figure 7: Representative example of the gating strategy of the flow cytometry analysis conducted on immune cells isolated from human blood and from murine U87 tumors in huNOG-EXL mice. Debris and doublets/aggregates were removed. Single cells were divided into human and murine CD45+ cells based on control staining from naïve mouse spleen (top rows) and human donor PBMC (bottom rows). Murine CD45+ cells were divided into CX3CR1+/Tmem119+ microglia and negative non-microglia. Non-microglia were

then divided into B220+ B cells and negative non-B cells. B220- non-B cells were divided into CD11b+ myeloid cells, and CD11c+/CD11b- dendritic cells. CD11b+ cells were divided into Mono-MDSC defined as Ly6C+/Ly6G, PMN-MDSC defined as Ly6G+, and TAM defined as Ly6C-/Ly6G-/F4-80+. Human CD45+ cells were divided into CD19+ B cells, CD3+ T cells, and double-negative non-B and non-T cells. CD19-/CD3- cells were divided into HLA-DR+ and HLA-DR- cells. HLA-DR- cells were divided into CD11b+/CD33+ MDSCs and CD11b+/CD33-/CD68+ TAM. MDSC were further divided into CD15+/CD14- PMN-MDSC and CD14+ Mono-MDSC. HLA-DR+ cells were divided into CD68+/CD11c- Macrophages, CD68+/CD11c+ monocyte-derived dendritic cells, and CD68-/CD11c+ dendritic cells.
