# Supplementary material for: STING agonist 8803 reprograms the immune microenvironment and increases survival in preclinical models of glioblastoma
Source: J Clin Invest. 2024 Jun 17;134(12):e175033. doi: 10.1172/JCI175033 (PMC11178548; doi:10.1172/JCI175033)
Supplement: Unedited blot and gel images [file jci-134-175033-s162.pdf]

Original blots for Figure 6C (represented by B and C) and 6D (represented by A).

A

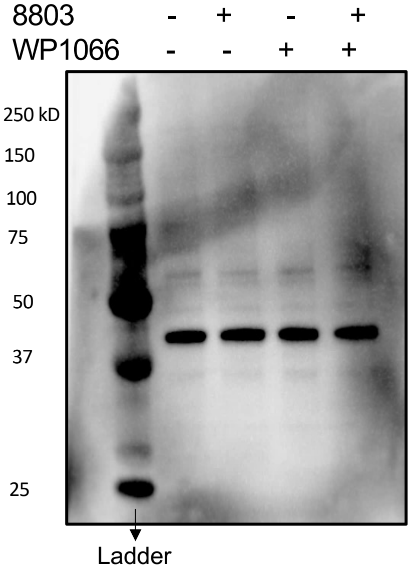

Actin

B

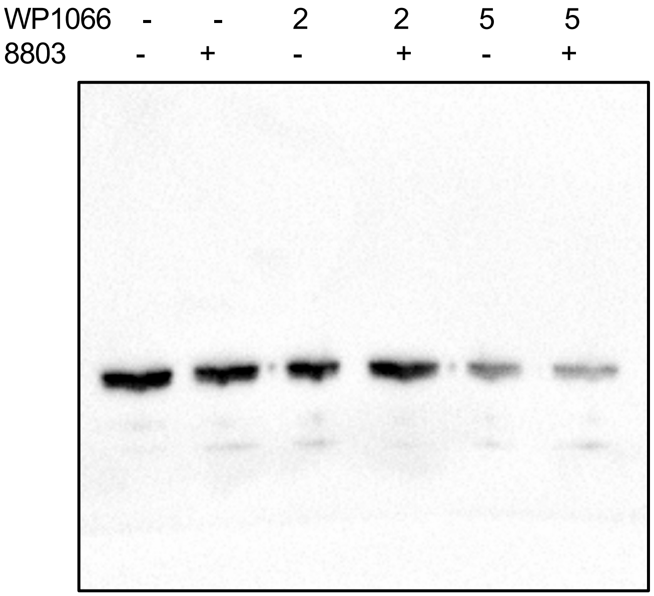

STING

C

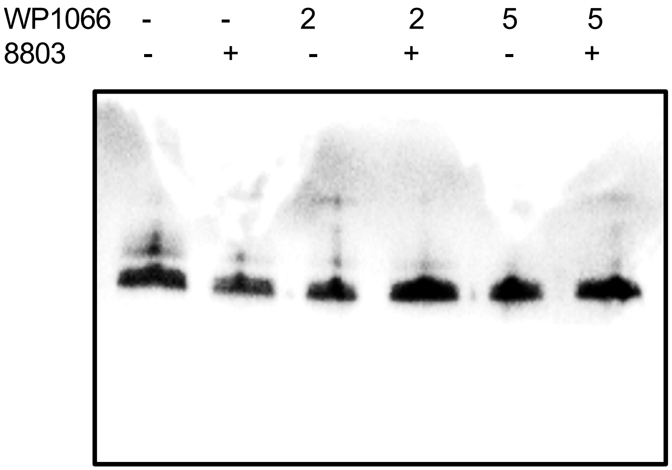

Actin

Human and Mouse Glioma Cell Lines original blots for Supplementary Figure 5A.

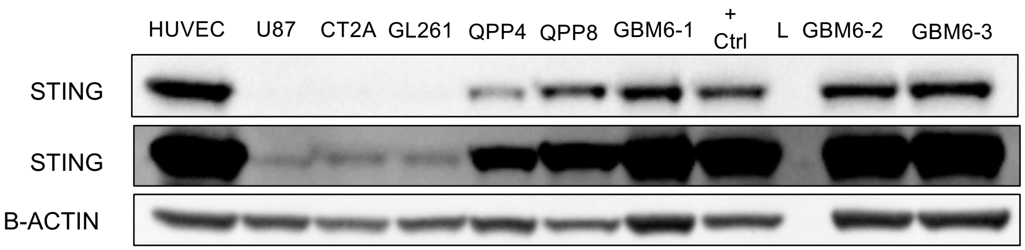

(1s999ms capture; no saturation on any band)

(2min capture; NOTE HUVEC, QPP4, QPP8, GBM6-1, GBM6-2, GBM6-3 bands are saturated)

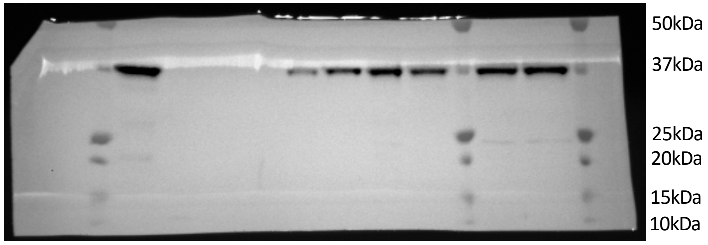

STING
(1s999ms capture)

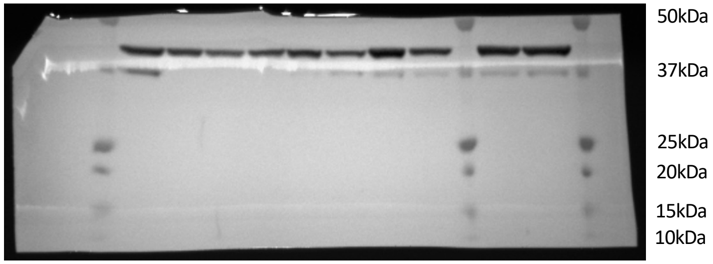

Beta-Actin

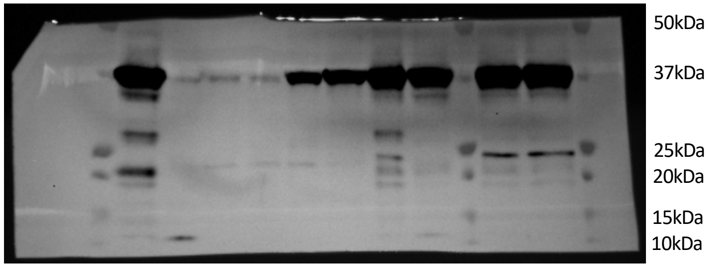

STING
(2min capture; NOTE HUVEC, QPP4, QPP8, GBM6-1, GBM6-2, GBM6-3 bands are saturated)
